# Secretion of Genome-Free Hepatitis B Virus – Single Strand Blocking Model for Virion Morphogenesis of Para-retrovirus

**DOI:** 10.1371/journal.ppat.1002255

**Published:** 2011-09-22

**Authors:** Xiaojun Ning, David Nguyen, Laura Mentzer, Christina Adams, Hyunwook Lee, Robert Ashley, Susan Hafenstein, Jianming Hu

**Affiliations:** 1 Department of Microbiology and Immunology, The Penn State University College of Medicine, Hershey, Pennsylvania, United States of America; 2 Division of Infectious Diseases, Department of Medicine, The Penn State University College of Medicine, Hershey, Pennsylvania, United States of America; University of Southern California, United States of America

## Abstract

As a para-retrovirus, hepatitis B virus (HBV) is an enveloped virus with a double-stranded (DS) DNA genome that is replicated by reverse transcription of an RNA intermediate, the pregenomic RNA or pgRNA. HBV assembly begins with the formation of an “immature” nucleocapsid (NC) incorporating pgRNA, which is converted via reverse transcription within the maturing NC to the DS DNA genome. Only the mature, DS DNA-containing NCs are enveloped and secreted as virions whereas immature NCs containing RNA or single-stranded (SS) DNA are not enveloped. The current model for selective virion morphogenesis postulates that accumulation of DS DNA within the NC induces a “maturation signal” that, in turn, triggers its envelopment and secretion. However, we have found, by careful quantification of viral DNA and NCs in HBV virions secreted *in vitro* and *in vivo*, that the vast majority of HBV virions (over 90%) contained no DNA at all, indicating that NCs with no genome were enveloped and secreted as empty virions (i.e., enveloped NCs with no DNA). Furthermore, viral mutants bearing mutations precluding any DNA synthesis secreted exclusively empty virions. Thus, viral DNA synthesis is not required for HBV virion morphogenesis. On the other hand, NCs containing RNA or SS DNA were excluded from virion formation. The secretion of DS DNA-containing as well as empty virions on one hand, and the lack of secretion of virions containing single-stranded (SS) DNA or RNA on the other, prompted us to propose an alternative, “Single Strand Blocking” model to explain selective HBV morphogenesis whereby SS nucleic acid within the NC negatively regulates NC envelopment, which is relieved upon second strand DNA synthesis.

## Author Summary

Hepatitis B virus (HBV), an important global human pathogen and the main cause of liver cancer worldwide, is classified as a para-retrovirus, as it replicates by reverse transcription, i.e., copying of RNA to DNA, like retroviruses. However, different from retroviruses that are RNA viruses replicating via a DNA intermediate, HBV is a DNA virus that replicates through an RNA intermediate. Like retroviruses, HBV initially packages an RNA copy of its genome into intracellular subviral particles. However, complete HBV virions contain only a double-stranded (DS) DNA. The long-standing model to explain this selective presence of DS DNA in HBV virions postulates that DS DNA synthesis is required to trigger virion secretion. We have found, however, that virion secretion does not require any DNA synthesis. Rather, the presence of the single-stranded RNA (or the single-stranded DNA intermediate of reverse transcription) negatively regulates virion formation. These results thus change the prevailing paradigm in understanding HBV morphogenesis and also have important implications for virus assembly in general. Furthermore, they raise the important question regarding the role of empty HBV virions identified here in viral replication and pathogenesis.

## Introduction

The hepatitis B virus (HBV) is a global human pathogen that chronically infects hundreds of millions and causes a million fatalities yearly. It belongs to the *Hepadnaviridae* family, which also includes several related animal viruses such as the duck hepatitis B virus (DHBV) [1]. Hepadnaviruses contain a small (ca 3 kb), partially double-stranded (DS) DNA genome enclosed within an icosahedral capsid that is formed by 240 (or 180 in a minority population) copies of the same viral protein, the core or capsid protein (HBc), and is, in turn, coated with an outer envelope. As pararetroviruses, hepadnaviruses assemble initially as immature nucleocapsids (NCs), packaging an RNA pregenome (pgRNA). These immature NCs undergo a process of maturation first to NCs containing a single-stranded (SS) DNA (still considered immature) and subsequently to mature NCs containing the DS DNA genome, via reverse transcription of pgRNA inside the maturing NCs. Only the mature NCs are then enveloped by the viral envelope or surface (HBs) proteins and secreted extracellularly [2], [3].

How genome maturation, within NCs, is coupled to envelopment, from without, remains poorly understood. In particular, the exact nature of the viral genome that is ultimately responsible for regulating virion secretion is not yet clear. As SS RNA or DNA is not secreted in virions but DS DNA, in either the major, relaxed circular (RC) or minor, double-stranded linear (DSL) form, or RNA-DNA hybrid, is [3]–[10], the prevailing model posits that the accumulation of DS DNA as a result of second strand elongation during reverse transcription triggers a structural change in the maturing NC that, in turn, signals envelopment and secretion [2], [3], [11], [12]. Thus, this so-called maturation signal would emerge on the mature NC only as reverse transcription approaches completion and positively regulate virion secretion.

On the other hand, it has been suggested that HBV may secrete virions containing no DNA at all. Two populations of HBV virion particles were found to circulate in the blood of infected patients decades ago, with one having a lighter buoyant density than the other [13]–[15]. These so-called “light” virion particles contained HBV envelope and core proteins but in contrast to the “heavy” particles, displayed no endogenous polymerase activity, which reflects DNA synthesis by the virion reverse transcriptase (RT) using the endogenous DNA template. These light particles also appeared empty under electron microscopy (EM) and were assumed to be devoid of viral DNA. However, these early reports did not directly determine the levels of viral DNA in the light virions or whether they contained viral RNA or host nucleic acid. A more recent study suggested that the light virion particles might actually contain, instead of the normal capsid protein, an aberrantly processed precore protein [16]. Another recent report found that small amounts of enveloped HBV capsids devoid of viral genome were secreted in transfected cell cultures but those were deemed aberrant [9], [16]. Thus, it has remained unclear if HBV does secrete DNA-free virions and if so, whether it is part of the normal virion morphogenesis process.

In our efforts to further define the nature of the viral genome that underlies selective NC envelopment and virion secretion, we have found that genome packaging or DNA synthesis, *per se*, was actually not required at all for virion secretion as HBV mutants able to form normal capsids but incapable of viral RNA packaging or DNA synthesis, due to defects in RT, were secreted readily as enveloped, empty (nucleic acid-free) virions. Furthermore, quantitative analyses of virion-associated DNA and capsids revealed that the vast majority of virions secreted by the wild type (WT) HBV in cell culture as well as in infected chimpanzees contained no viral DNA. We propose a new model to explain the selective HBV virion morphogenesis that can reconcile the seemingly contradictory observations that virion formation selects stringently for DS, and against SS, nucleic acid genome and yet empty HBV virions devoid of any nucleic acid are secreted.

## Results

### Dramatic reduction of HBV DNA synthesis did not decrease virion secretion

**Figure 1:**
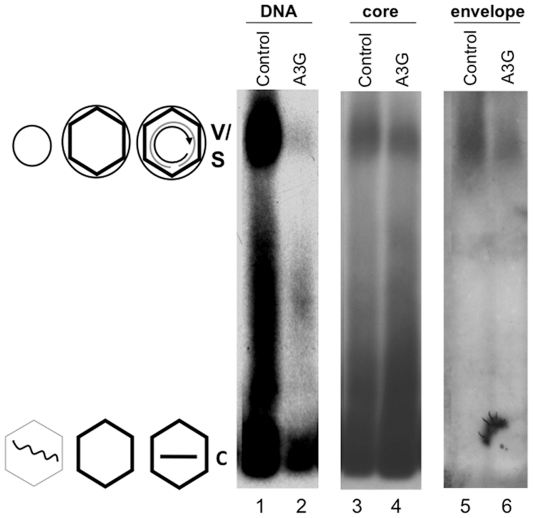
Inhibition of HBV DNA synthesis by Apobec3G did not block virion secretion. Culture media from HepG-2 cells transfected with pCMV-HBV plus pA3G-Flag (lanes 2, 4, 6; A3G or Apobec3G), or plus vector control (lanes 1, 3, 5; control) were collected and viral particles concentrated from the medium were resolved by agarose gel electrophoresis and transferred to nitrocellulose membrane. Viral DNA (lanes 1–2), and the core (lanes 3–4) and envelope (lanes 5–6) proteins were detected sequentially using a ^32^P-labeled HBV DNA probe, the anti-core antibody, and the anti-S antibody respectively, all on the same piece of membrane. V, virions, containing either RC DNA or empty; C, naked capsids, containing DNA (mostly SS), RNA, or empty. The diagrams on the side depict the structures of the virions and capsids. Wavy line, viral RNA; straight single line, SS DNA; concentric partial circle, RC DNA; hexagon, capsid; large circle, virion envelope; small circle, HBsAg particles (S, containing no capsid nor genome) comigrating with the virions.

**Figure 2:**
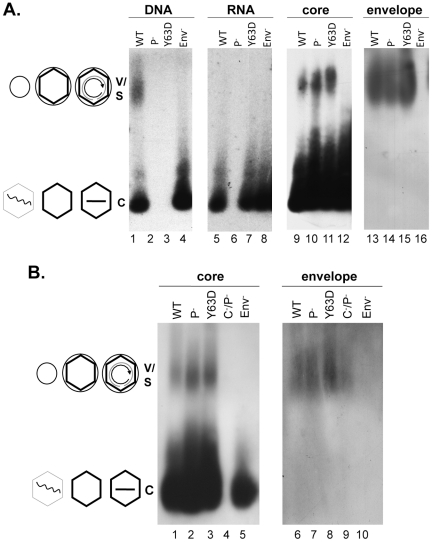
Analyses of HBV virion secretion by native agarose gel electrophoresis. WT or mutant HBV genomes (Pol^-^, defective in pgRNA packaging; Y63D, competent in pgRNA packaging but defective in DNA synthesis; Env^-^, envelope deletion; C^-^P^-^, deficient for both core and polymerase expression) were transfected into HepG-2 cells. Viral particles concentrated from the culture medium were resolved by agarose gel electrophoresis and transferred to nitrocellulose membrane. Viral DNA (**A**, lanes 1–4) or core (**A**, lanes 9–12; **B**, lanes 1–5) and envelope proteins (**A**, lanes 13–16; **B**, lanes 6–10) were detected as in **Figure 1**. Viral RNA (**A**, lanes 5–8) was detected as for viral DNA, except that the samples were resolved on a separate gel that was not treated with NaOH prior to transfer and the membrane probed with a plus-strand specific riboprobe. V, virions, containing either RC DNA or empty; C, naked capsids, containing DNA, RNA, or empty. The diagrams on the side depict the structures of the virions and capsids as described in **Figure 1**.

We obtained the first hint that HBV virion secretion may not necessarily require viral DNA synthesis during our recent studies on the anti-HBV effect of the cellular antiviral protein, Apobec3G (A3G) [17]. As shown in **Figure 1** and reported earlier [18], over-expression of A3G led to a dramatic reduction of HBV DNA associated with non-enveloped (naked) NCs as well as virions secreted into the culture medium of transfected human hepatoma (HepG-2) cells, which were resolved by native agarose gel electrophoresis [19]. However, we found, unexpectedly, that the amount of enveloped capsids secreted into the culture medium was not decreased at all by A3G. The identity of the virion signal was further verified by its association with, and dependence on, the viral envelope proteins (**Figures 1** & **2**) and its authentic buoyant density (see below). Naked capsids, which are routinely released into cell culture medium via an unknown mechanism unrelated to virion secretion and are of uncertain significance [20], were not affected by A3G though their DNA content was reduced as reported before [18]. HBsAg particles, which contain only the viral envelope proteins but no capsids nor genome [1], were not affected by A3G either (**Figure 1**). As HBsAg particles are normally released in large excess (100-fold or more) over virions [1], the surface protein signals detected in **Figures 1** and **2** were virtually all from the HBsAg but the virion and HBsAg comigrated on the gel. This was also evidenced by the surface protein signal migrating at the virion position from a mutant defective in core and polymerase protein expression (C^-^P^-^) and thus could only secrete HBsAg particles but no virions (neither empty nor DNA-filled) at all (**Figure 2B**). These results indicated that viral DNA synthesis might not be required for NC envelopment and virion formation, in apparent contradiction to the prevailing maturation signal hypothesis.

### HBV mutants defective in RNA packaging or DNA synthesis secreted enveloped capsids containing no viral RNA or DNA

To further examine the relationship between genome content and virion secretion in HBV, we determined the virion secretion capacity of two mutants, Pol^-^ and Y63D; Pol^-^ is defective in polymerase expression and unable to package viral RNA [21] whereas Y63D is defective in DNA synthesis but fully functional in RNA packaging [22] (**Figure 2**). Native agarose gel analyses of HBV particles concentrated from the culture medium of transiently transfected HepG-2 cells showed that as reported before [5], pgRNA was not found in enveloped virions though readily detectable in naked capsids of either WT or the Y63D mutant (**Figure 2**). As anticipated, no viral RNA was detected in either NC or virion particles from the Pol^-^ mutant, and only the WT, but not the Y63D or Pol^-^ mutant, contained viral DNA in virions or naked capsids. However, capsid protein signal was clearly detected not only with the naked capsids but also with the virion particles from the Y63D and Pol^-^ mutants, at levels at least as high as those of the WT. As a negative control, the Env^-^ mutant did not show any capsid or DNA signal migrating at the virion position.

**Figure 3:**
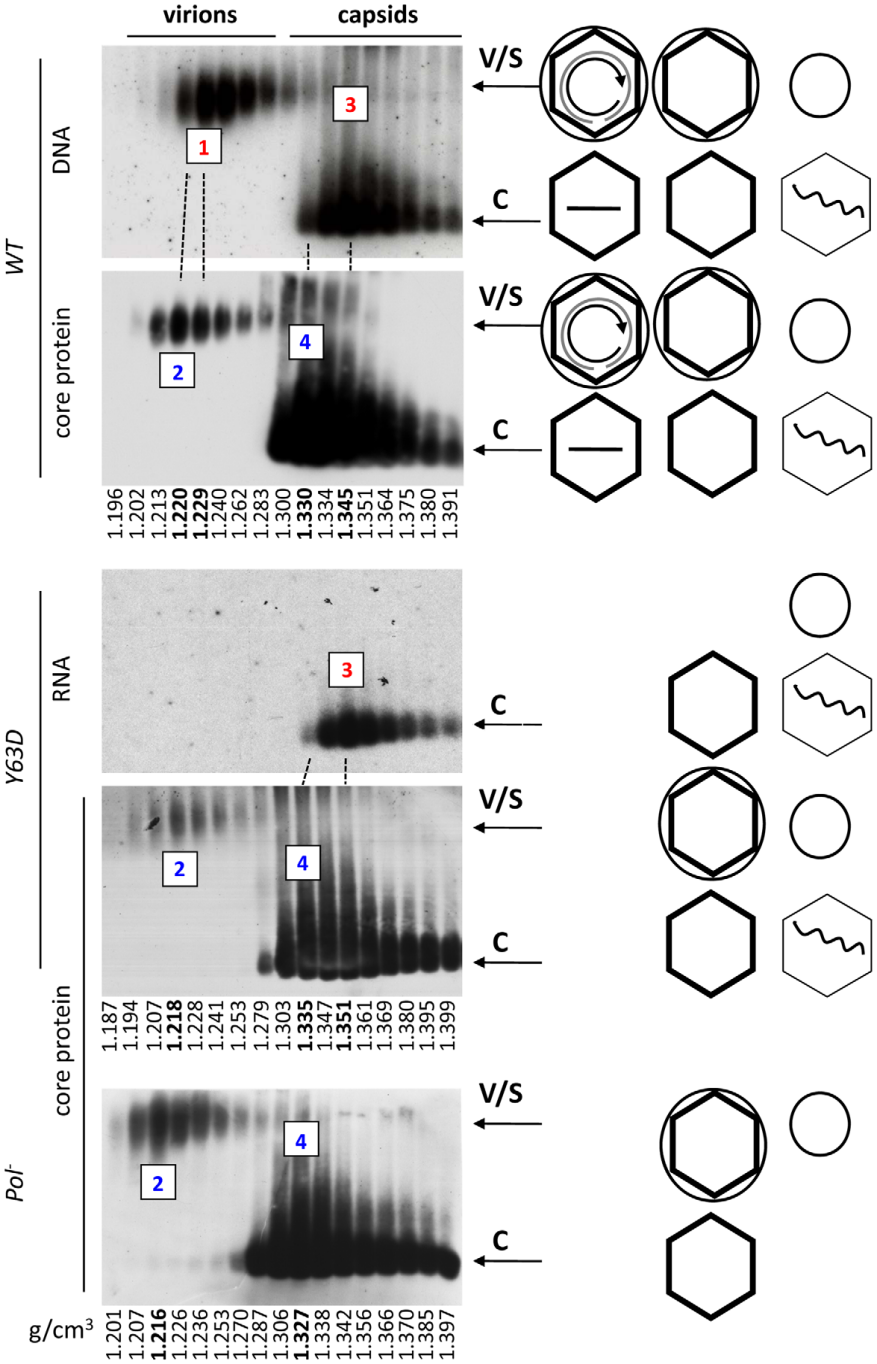
Analyses of HBV virion secretion by CsCl density gradient analyses. WT or mutant HBV genomes were transfected into HepG-2 cells and viral particles concentrated from the culture media as in **Figure 1**. The concentrated media were then fractionated by CsCl gradient centrifugation. Gradient fractions were analyzed by resolving viral particles on native agarose gels. HBV DNA (panel 1), RNA (panel 3), and core protein (panels 2, 4, 5) were detected as described in **Figures 1** & **2**. WT, panels 1 & 2; Y63D, panels 3 & 4; Pol^-^, panel 5. The numbered fractions mark the DNA-containing (#1) or DNA-free (#2) virion peaks, and the naked capsid peaks containing viral DNA or RNA (#3) or no nucleic acid (empty, #4), with their respective buoyant densities indicated in bold at the bottom. Note that the first two panels from the top were derived from the same membrane sequentially probed for viral DNA and the core protein, and the 3^rd^ and 4^th^ panels also from the same membrane sequentially probed for viral RNA and core protein. The dashed lines are used to align the identical lanes from the same membrane. The diagrams on the side depict the structures of the virions and capsids as described in **Figure 1**.

The co-migration of the putative Y63D and Pol^-^ virions with the WT virions as well as with the envelope signals supported their identification as authentic virions, which was further verified by density gradient centrifugation (**Figure 3** **; Figure S1**). Native agarose gel electrophoresis analyses of viral particles fractionated on the CsCl gradient confirmed that enveloped capsids, i.e., virions, judged both by their density profile and mobility on the agarose gel, were indeed secreted by the Y63D and Pol^-^ mutants even though they contained no viral DNA. Consistent with the notion that these enveloped capsids contained no nucleic acid (viral or cellular; also see below) at all, the empty virions (marked as peak #2) produced by the Y63D or Pol^-^ mutant had a slightly lower density (as predicted by their lack of nucleic acid) than the authentic, DNA-containing virions secreted by the WT HBV (peak #1). Furthermore, the capsid signal peak (marked as #2) from the WT virion fractions also had a lower density than the DNA peak. This later result suggested that most of the WT HBV virions might also be empty such that the bulk virions (empty) had a density lighter than the minor amounts of DNA-containing virions.

**Figure 4:**
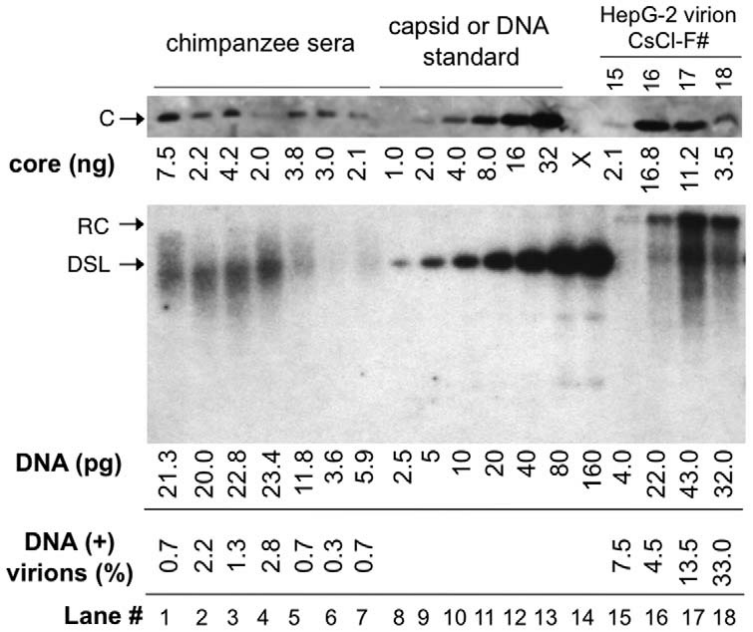
Quantitative analyses of DNA-filled and empty virions secreted by WT HBV *in vitro* and *in vivo*. HBV virions in the sera of experimentally infected chimpanzees (lanes 1–7; 2 µl each serum) and purified from WT HBV transfected HepG-2 cells by CsCl density gradient centrifugation (lanes 15–18; 30 µl each fraction), along with known amounts of HBV capsid standard (1–32 ng; lanes 8–13), were resolved by SDS-PAGE; Virion-associated core protein was detected by western blot analysis using the anti-HBV core monoclonal antibody (top image). The virion DNA released by SDS-protease digestion from the same chimpanzee sera (lanes 1–7; 2 µl each serum) and HepG-2 virion fractions (lanes 15–18; 2 µl each fraction) were also resolved on an agarose gel, along with known amounts of HBV DNA (3 kb, 2.5–160 pg, lanes 8–14), and detected by Southern blot analysis (bottom image). Lane 1 was from chimpanzee A0A006 at week 7 post-infection (PI); lanes 2 & 3, chimpanzee 1603 at week 15 and 16 PI, respectively; lanes 4 & 5, chimpanzee 1618 at week 14 and 17; lanes 6 & 7, chimpanzee 1616 at week 20 and 23 PI. The amounts of HBV core protein (top) and virion DNA (bottom) are indicated at the bottom of the lanes. The percentages of DNA-containing virions, calculated as the molar amounts of HBV DNA divided by the molar amounts of HBV capsid (240 copies of core subunits per capsid) and multiplied by 100, are also indicated. Please note that 15-fold more HepG-2 virions were loaded on the SDS-PAGE gel than on the agarose gel, which was taken into account in the calculation of DNA-containing virions. F#, virion fraction number from the CsCl gradient. C, core protein; RC and DSL, RC and DSL DNA; X, blank lane (lane 14) on the SDS-PAGE gel.

Although small amounts of naked NCs migrated near the position of the virions after the CsCl gradient fractionation step, presumably in aggregated forms as a result of exposure to the high salt concentration of the gradient as suggested earlier [9], the naked NCs were clearly separated from the virions by the CsCl density gradient fractionation such that there was no naked NC contamination in the virion fractions (**Figure 3**). In addition, consistent with numerous previous reports (see Introduction), immature SS DNA was found in naked NCs but not in the virion fractions (**Figure S2**; and also **Figure 4** below), again indicating clear separation of virions from naked NCs. On the other hand, no naked capsid signal was detected near the virion position on the agarose gel without the CsCl gradient centrifugation step as indicated by the absence of any capsid protein or viral DNA signal at the virion position from an HBV mutant defective in envelope protein expression (**Figure 2A**, lanes 4, 12; **Figure 2B**, lane 5). Furthermore, abolishing viral envelope protein expression eliminated any capsid or viral DNA signal in the virion fractions on the CsCl gradient (**Figure S3**), again indicating the secretion of the empty virions, just like the DNA-containing virions, was absolutely dependent on the viral envelope proteins and no naked capsids contaminated the virion fractions following the density gradient fractionation.

### WT HBV secreted mostly empty virions *in vitro* and *in vivo*

To follow up on the above suggestion, we decided to quantify the amount of HBV DNA and the capsid protein signals within the virion fractions secreted by WT HBV transfected HepG-2 cells. This revealed that most secreted HBV virions (from 92.5% in the lighter fraction to 67% in the heavier fraction) from transfected cells (**Figure 4**, lanes 15–18) were indeed devoid of any viral DNA. These estimates were based on quantifications of the levels of virion-associated capsids (based on 240 copies of core protein per capsid) vs. the virion DNA. Although it is theoretically possible that the DNA-free virion capsids may have reacted differently than the DNA-containing virion capsids with the anti-HBc antibody used for the western blotting, this was made unlikely by the fact that the relative signals of the capsid proteins remained constant across the lighter (virtually DNA-free) and heavier (with more DNA-containing virions) fractions (**Figure S4**), whether the capsid protein levels were estimated as native particles resolved on an agarose gel or as denatured subunits resolved by sodium dodecyl sulfate-polyacrylamide gel electrophoresis (SDS-PAGE) and detected by either a polyclonal antibody or a monoclonal directed at a linear epitope at the N-terminus of the capsid protein [23].

**Figure 5:**
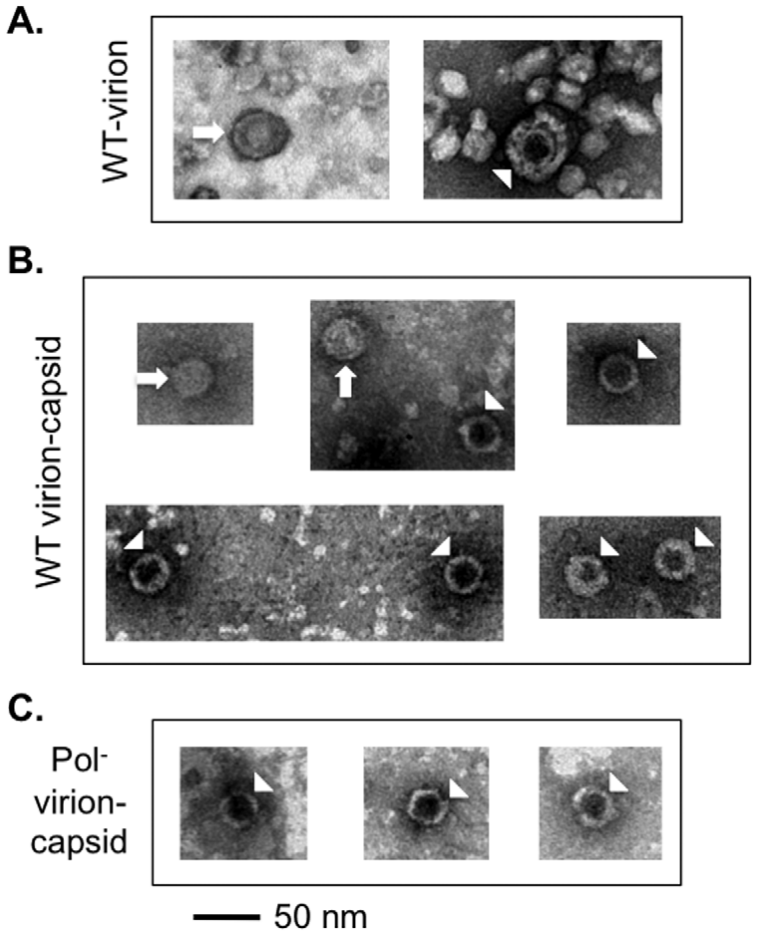
EM of HBV virions and virion-derived capsids. HBV WT or Pol^-^ virions released from transfected HepG-2 cells were fractionated by CsCl density gradient centrifugation. The purified WT virions (**A**), or capsids released from the WT (**B**) or Pol^-^ (**C**) virions by NP40 treatment were visualized by EM after negative staining. The arrows indicate the filled virion or virion-derived capsids and the arrowheads denote the empty virion or virion-derived capsids. Note that the smaller particles in **A** represent the HBsAg spheres, which were much more numerous than the virions and were removed by the detergent treatment in **B** and **C**.

**Figure 6:**
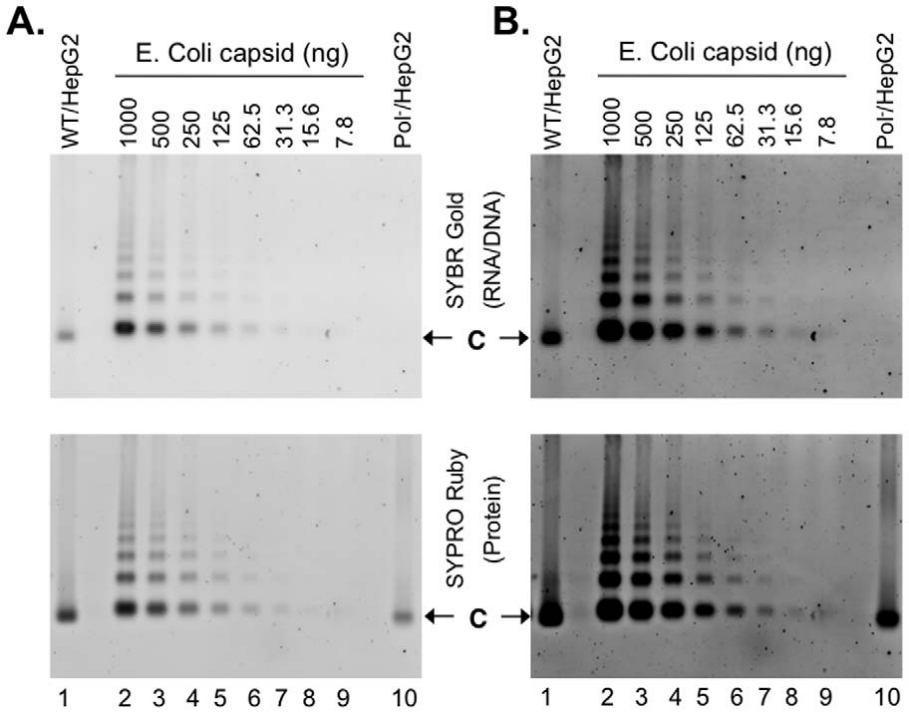
Nucleic acid and protein staining of intracellular capsids. WT HBV NCs (lane 1) and HBV capsids devoid of the polymerase (Pol^-^) (lane 10) were purified from HepG-2 cells transiently transfected with pCMV-HBV and pCMV-HBV-Pol^-^, respectively. WT HBV capsids purified from E. Coli (lanes 2–9) were purchased from Virogen. The capsids were resolved by native agarose gel electrophoresis. Capsid-associated nucleic acid was detected by SYBR Gold staining (top) and the capsid proteins were detected by destaining of the SYBR Gold signal and subsequent restaining of the same gel with SYPRO Ruby (bottom). Note that **A** & **B** are from the same gel and the images in **B** are contrast-enhanced to display the weak signals. C, capsids.

**Figure 7:**
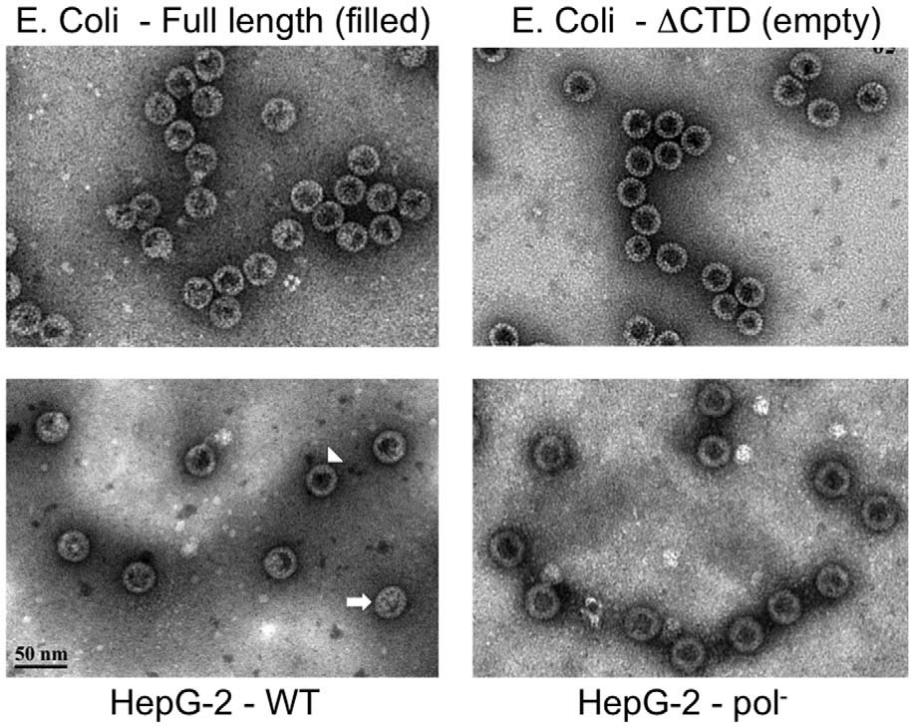
EM of intracellular capsids. Native HBV NCs purified by sucrose gradient centrifugation from transfected HepG-2 cells (WT, bottom left; Pol^-^, bottom right) were visualized by EM after negative staining with phosphotungstic acid. E. Coli-derived HBV WT (top left) or C-terminally truncated (at position 144, C144 or ΔCTD; top right) capsids were examined in parallel as (RNA) filled and empty capsid controls, respectively. Two WT capsids with distinct appearances, empty (arrowhead) and filled (arrow), are indicated.

As reported earlier [13], [14] using HBV positive human sera, two kinds of WT HBV virions were observed by EM after negative staining, with either a filled or empty looking inner capsid under EM (**Figure 5A**). However, for most virions, the stain did not penetrate the envelope thus making it difficult to discern if the capsids within the virions were filled or empty. To visualize better the capsids contained in the virions, the virion envelope was removed by detergent lysis [12], [14] and the released capsids were observed under EM. Upon removal of the virion envelope by the detergent treatment, we found that the majority of the released capsids (ca 70%) had an empty appearance consistent with their containing no nucleic acid while the remaining showed a filled appearance presumably containing the DS DNA (**Figure 5B**). We also released the capsids from the Pol^-^ mutant virions using the same method and found that all released Pol^-^ capsids showed the empty appearance, consistent with their lower density and containing no nucleic acid (**Figure 5C**). As higher concentrations of capsids were obtainable from intracellular sources, more detailed analyses of the empty and filled capsids, including 3-D image reconstruction, were carried out on those capsids and are described below (**Figures 6** & **7**; **Figures S8** & **S9**).

Intrigued by these results, we decided to quantify the levels of viral DNA and capsid protein in the sera of HBV infected chimpanzees [24]. As shown in **Figure 4** (lanes 1–7) and **Figure S5**, the vast majority (97.8–99.7%) of HBV virions found in the sera from all four infected chimpanzees harvested at two different times post-infection contained no viral DNA. HBV virions in one of the chimpanzee sera were also fractionated by CsCl density gradient centrifugation. As with the virions released by transfected cells in culture, the core protein peak (**Figure S5B**, lane 2) in the serum virions had a lighter density than the DNA peak (**Figure S5B**, lane 4), indicating again the majority of the WT virions in the serum were DNA-free and had a lighter density. The core protein signal in the DNA peak fraction (lane 4) was below the limit of detection since our core protein Western blotting had a sensitivity of ca 1 ng/lane while the Southern blotting had a sensitivity of less than 2.5 pg/lane (**Figure 4**). The virion DNA secreted by the infected chimpanzees in vivo migrated mostly just below the 3 kb DS DNA standard (**Figure 4**, bottom image, lanes 1–7), typical for the incompletely DS HBV virion RC DNA isolated from infected patients [10], [25]. For reasons yet to be understood, HBV virion DNA secreted by the transfected hepatoma cells in vitro appeared more fully DS [26], migrating above the 3 kb linear DNA (**Figure 4**, bottom, lanes 15–18).

As the HBsAg particles are present in large excess (100–1,000 fold or more) over virions [1] and we detected also a large excess of empty virions over DNA-containing ones (with total virion core proteins reaching 1–3.75 µg/ml or 1.6–4.4×10^11^ virions/ml), it was of interest to estimate the levels of HBsAg in the sera from the infected chimpanzees. To this end, purified recombinant HBsAg (eEnzyme) was used a quantitative standard for western blotting. Due to the undefined oligomeric states (a mixture of monomers, dimers and higher order oligomers; eEnzyme) of the HBsAg standard, it was resolved by SDS-PAGE along with different dilutions of two serum samples from the same chimpanzee, one before and one after HBV infection. The surface proteins were detected using a rabbit polyclonal antibody that can recognize the denatured HBsAg (**Figure S6A**) and the HBsAg concentration in the HBV-positive serum was estimated based on comparison with the standard. The HBsAg concentrations in the other chimpanzees were then estimated by comparison to this serum after they were resolved together on an agarose gel (**Figure S6B**). Our estimates of the serum HBsAg levels in all four infected chimpanzees ranged from 124–1,899 µg/ml, or ca 3×10^13^–4.4×10^14^/ml assuming 100 copies of the small surface protein per HBsAg sphere [1], [27]. Thus, the HBsAg levels were ca 167- to 1,688-fold above the virions (both DNA-containing and empty). These estimates were in good agreement with earlier estimates of HBsAg concentrations in the serum of HBV infected human patients of 10^13^/ml or 40 µg/ml [28] to more recent quantifications of up to 10^15^ or 4 mg/ml [29]–[31].

### Empty HBV capsids were assembled in human cells that did not package non-specific RNA

The secretion of HBV virions containing no nucleic acid at all implied that empty HBV capsids, containing no viral or cellular nucleic acid, were assembled in host cells. In support of this, the naked capsids harvested from the WT and Y63D transfections peaked at a lower density (**Figure 3** & **Figure S1**, peak #4;) than the viral RNA or DNA signals (i.e., filled NCs) that peaked at a higher density (peak #3). Although this capsid protein peak contained some viral RNA or DNA, it likely represented the overlap from the heavier, nucleic acid-containing NCs; earlier (still lighter) capsid fractions contained little to no viral RNA or DNA. The naked capsids of the polymerase-null mutant, being devoid of any viral RNA or DNA **(** **Figure 2**) or cellular nucleic acid (see **Figure 6** below), peaked at the same light density as the capsid protein peak (#4) from the WT and the Y63D mutant, lighter than the RNA or DNA-containing NCs peak (#3).

To further verify that empty HBV capsids, containing no viral or cellular nucleic acid, were indeed made in cells, we purified intracellular capsids from transfected HepG-2 cells by sucrose gradient centrifugation, resolved them on agarose gels along with HBV capsids purified from E. Coli that are known to be filled with nonspecific RNA [32]–[34], and detected the capsids (the protein signal) or their associated nucleic acid with the highly sensitive general protein (SYPRO Ruby) or nucleic acid (SYBR Gold) stain. The detection sensitivity of the capsid-associated RNA (approximately 8 ng capsids, or 1.6 ng RNA assuming each capsid packages 3 kb RNA; **Figure 6**) was about 5-fold less than that of purified RNA (approximately 0.3 ng RNA; **Figure S7**), presumably due to the sequestration of RNA inside the capsids. Therefore, instead of attempting to quantify the absolute amounts of nucleic acid associated with the HepG-2 cell-derived capsids, we used the E. Coli-derived capsids, not purified RNA or DNA, for relative comparison.

The HBV Pol^-^ capsids did not show any detectable nucleic acid signal (**Figure 6**, top, lane 10), as anticipated. Given that 244 ng of Pol^-^ capsids (**Figure 6**, bottom, lane 10), estimated based on comparison to the E. Coli-derived capsid standard (lanes 2–9), was loaded and the RNA associated with 7.8 ng bacterially derived capsids was detectable, the amount of RNA in the pol^-^ capsids, if any, was thus less than 3% of that in the E. Coli-derived capsids. The capsids harvested from WT HBV transfected HepG-2 cells (**Figure 6**, lane 1) were found to contain detectable but much less (ca 35%) nucleic acid when compared to the E. Coli-derived capsids. Thus, the amount of WT HBV capsids purified from HepG-2 cells loaded was 707 ng **(** **Figure 6**, bottom, lane 1) but its nucleic acid content was equivalent only to 269 ng E. Coli-derived capsid **(** **Figure 6**, top, lane 1). Titration of the WT HBV capsids harvested from HepG-2 cells showed that the nucleic acid content could be detected by the staining method when ca 100 ng of capsid was loaded per lane (**Figure S8**). As the nucleic acid signal detected in the WT HBV NCs was a mixture of capsid-associated viral DNA as well as RNA, and the SYBR Gold stain had a higher (by ca 7 fold) detection sensitivity for DNA than RNA (**Figure S7**), the amount of nucleic acid associated with the WT capsids from HepG-2 cells, relative to that associated with the bacterially-derived capsids that contain only RNA, were likely overestimated. Thus, these results clearly indicated that the majority of WT HBV capsids assembled in hepatoma cells were also empty.

The purified capsids were further subjected to negative staining and EM (**Figure 7**), which showed that most (ca 80–90%) (**Table S1**) HBV capsids harvested from the WT HBV transfected HepG-2 cells, and virtually all capsids from the HBV Pol^-^ transfected cells, displayed the thin-walled or empty appearance characteristic of nucleic acid-free capsids such as the empty, C-terminally truncated HBV capsids purified from E. Coli [15], [35], [36]. The rest (ca 10–20%) of the WT HBV capsids from HepG-2 cells showed the thick-walled or filled appearance consistent with their containing RNA or DNA, similar to the full-length capsids derived from E. Coli that are known to contain non-specific RNA.

To obtain further information about the interior contents of the various HBV capsid populations, 3-D capsid maps were reconstructed from selected negatively stained particles from the EM images (**Table S1**). To check how the 3-D reconstruction program translated the EM images into a 3-D model, the WT HBV capsids from the HepG-2 cells were separated into two groups, those that appeared to be empty and those that appeared to be filled, and reconstructions were performed separately for each group. The empty group resulted in a map with a hollow core, and the filled group depicted electron densities inside of the capsid (**Figure S9**). In comparison, the 3-D map of the HBV Pol^-^ capsids also had a hollow core, in agreement with the results above showing that these capsids contained no detectable nucleic acid (**Figures 2** & **6**; **Figure S8**). Therefore, the results indicated that a 3-D reconstruction from negatively stained EM images could help distinguish between empty and filled viral particles. Capsids assembled from the full-length and C-terminally truncated capsid proteins expressed in E. Coli were also examined. The 3-D map of the full-length capsids from bacteria showed electron densities protruding inwards from the shell again consistent with the fact they contained non-specific RNA. In contrast, the C-terminally truncated capsids that appeared empty in the EM image also showed a hollow core in the corresponding 3-D model. These results agreed well with previously published cryo-EM structures that revealed that the amount of the RNA content in the E. Coli-derived HBV capsids depends on the length of the C-terminal nucleic acid binding domain of the core proteins [37].

A previous report suggested that an aberrant precore protein, lacking the C-terminal nucleic acid binding region of the core protein but retains the precore region preceding the core protein including the signal peptide, could assemble into abnormal capsids that were enveloped and secreted [16]. The constructs we used here to express the HBV core protein lacks the coding sequence for part of the precore region including the precore initiation codon and thus is not expected to produce any precore protein [38]. Also, the core protein in the purified capsids or virions migrated on SDS-PAGE as the full-length core protein standard (**Figure 4**; **Figures S4** & **S5**), suggesting that no truncation or degradation occurred. To confirm directly that the HBV core protein made in transfected cells indeed contained the C-terminal region, we employed an antibody specific for the last 14 residues of the core protein [39]. As anticipated, this antibody failed to detect a C-terminally truncated core protein but could detect the core protein in both the WT and Pol^-^ capsids; an antibody specific for the N-terminal sequence of the core protein detected the full-length as well as the truncated core proteins (**Figure S10**). These results thus verified that the HBV core protein expressed in our system was full-length and no aberrantly processed core or precore proteins were made.

## Discussion

Hepadnaviruses select only mature, DS DNA-containing, but not immature, RNA- or SS DNA-containing NCs for envelopment and extracellular secretion as virions. The molecular mechanism underlying this selective virion morphogenesis, which is a defining characteristic of all hepadnaviruses as pararetroviruses, remains to be elucidated. We have provided here multiple, complementary lines of evidence to demonstrate the secretion of empty HBV virions with no viral or cellular nucleic acid, i.e., enveloped capsids with no RNA or DNA. First, the secretion of empty virions, like the DNA-containing ones, depended on the expression of the viral envelope proteins as viral mutants defective in envelope expression did not secrete any virions, empty or DNA-filled. Second, the co-migration of the capsid and envelope proteins on the agarose gel and their co-fractionation on the density gradient indicated the capsids detected at the virion position were enveloped, as confirmed by EM observation. Third, the buoyant density of the empty virions were very close to the DNA-containing virions and much lower than the naked NCs, as expected for enveloped capsids, Fourth, the absence of viral DNA or RNA in the empty virions was shown by the secretion of virions by the Pol^-^ mutant that is incapable of packaging viral RNA or synthesizing DNA and by the Y63D mutant that can package pgRNA but can't synthesize any DNA, and by the lack of detection of any viral RNA or DNA by Southern blotting in these virions. Fifth, the absence of any nucleic acid, viral or cellular, in the empty virions was indicated by their slightly but reproducibly lower density than the DNA-containing virions and the EM observation of virions containing empty capsids as well as empty (mostly for the WT and entirely for the Pol^-^ mutant) capsids released from the virions. Sixth, the naked (non-enveloped) capsids from the WT were shown to be mostly, and those from the Pol^-^ HBV entirely, empty, with no viral or cellular nucleic acid. This was demonstrated by the absence of viral RNA or DNA by Southern blot analyses, by EM observation and 3-D image reconstruction, by their expected lighter density on the CsCl gradient, and by direct measurement of total nucleic acid in the capsids using sensitive nucleic acid staining methods. Thus, in the case of the Pol^-^ virions, it would have been very difficult to imagine that the secreted virions would have contained any RNA or DNA since the intracellular Pol^-^ capsids, which were the substrates for envelopment and virion formation, contained no detectable nucleic acid. Also, given that (a) most of the WT intracellular capsids contained no viral or cellular nucleic acid and the remaining WT capsids contained either viral RNA or DNA, (b) the intracellular capsids were the precursors to the enveloped virions, and (c) viral RNA-containing capsids were excluded from envelopment, it would have been difficult too to explain how the DNA-negative WT virions would have contained any nucleic acid.

**Figure 8:**
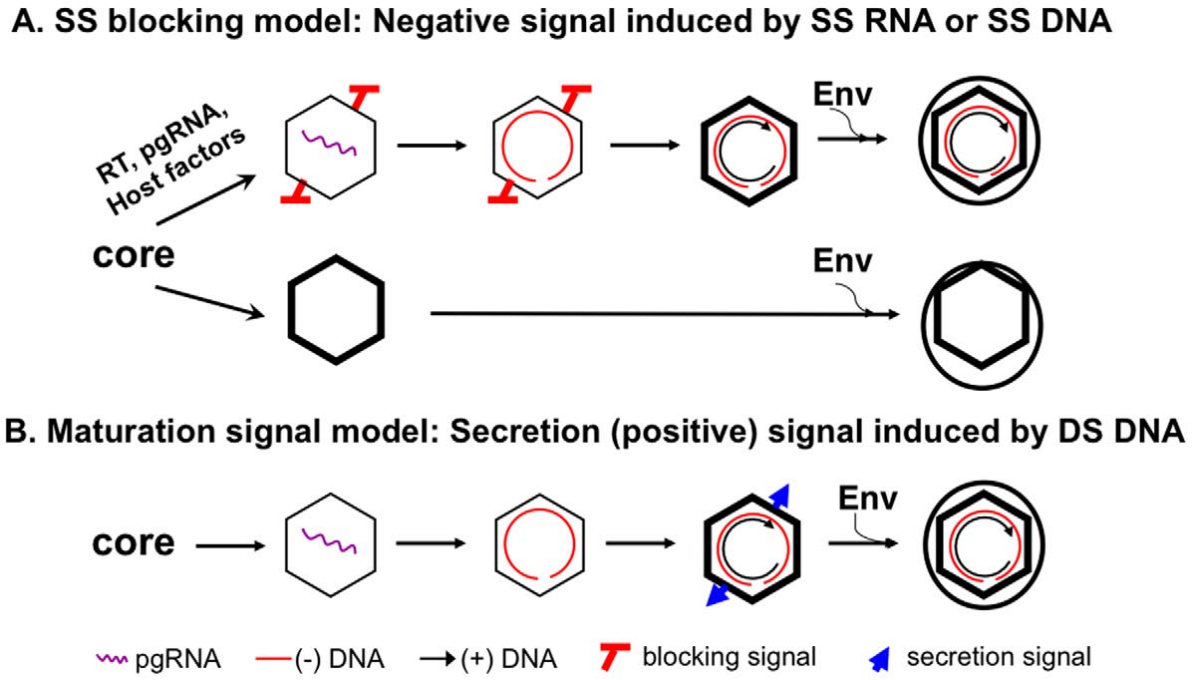
The single strand blocking hypothesis for selective hepadnavirus virion morphogenesis. The new model is presented in panel **A**, in comparison with the classical maturation signal model depicted in panel **B**. See Discussion for details.

The secretion of enveloped capsids, without any nucleic acid inside, is incompatible with the prevailing model of hepadnavirus morphogenesis, whereby virion formation requires DS DNA synthesis, i.e., NC maturation. Instead, we propose here that the paradox, i.e., the seemingly stringent selection of DS DNA over RNA or SS DNA in virion formation on one hand and yet secretion of empty HBV virions devoid of any nucleic acid on the other, can be resolved by invoking a new model, which we call “single strand (SS) blocking.” In this, negative signal model, the presence of SS DNA or pgRNA in the immature NCs actively prevents their envelopment by triggering a signal that negatively regulates NC envelopment (**Figure 8A**). The requirement of DS DNA for efficient virion secretion would NOT be due to a need to accumulate a threshold amount of total nucleic acid with the resulting increase in internal pressure, negative charge, genome rigidity, or some other unknown effect, which in turn would trigger a structural change (i.e., the maturation signal) on the maturing NC leading to envelopment and virion secretion, as envisioned by the classical maturation signal model (**Figure 8B**) [2], [3], [11], [12]. Rather, the apparent requirement for DS DNA synthesis in virion secretion reflects the need to remove the pgRNA or SS DNA, the trigger of the blocking signal, so as to relieve its inhibition on NC envelopment. Those capsids that do not package pgRNA (nor any other RNA, see below) would not display this negative signal and are thus competent for envelopment and secretion (as empty virions) (**Figure 8A**). Although the secretion of empty virions could be explained, in theory, by invoking a second envelopment signal for the empty capsids, independent from that emerging on the DS DNA-containing capsids, the simplest explanation is that the empty capsids and the DS DNA-containing capsids share the same envelopment signal.

The SS blocking model also predicts that decreasing the DS DNA (thus the total amount of nucleic acid inside the NC) may not block virion secretion. In support of this model, internally deleted short (ca 2 kb or shorter) DNAs, derived from packaging and reverse transcription of spliced HBV pgRNA have been detected commonly in the blood of HBV-infected patients [40], [41] and in transfected cell culture medium [42]–[44]. In contrast, the secretion of these shortened genomes in virions would be more difficult to explain with the classical maturation signal model.

The secretion of empty HBV virions requires assembly of empty capsids inside cells (**Figure 8A**). Three lines of evidence support the existence of these empty capsids: (1) their lower buoyant density than the nucleic acid-containing NCs, (2) lack of detection of nucleic acid with highly sensitive, general nucleic acid stain, and (3) their empty appearance under EM observation and 3-D capsid image reconstruction. HBV capsids assembled in insect cells did not package any cellular RNA either [45], [46] and presumably empty HBV capsids (so-called light cores) were also detected in the liver of HBV infected patients [14], [15], [47]. Thus, in contrast to capsids assembled in bacteria which package non-specific RNA [32]–[34], HBV capsids assembled in insect and mammalian cells do not package non-specific RNAs and are empty if they fail to package the viral pgRNA. The underlying mechanism for this difference remains to be elucidated but may be at least partly due to capsid phosphorylation in mammalian and insect cells that decreases its RNA binding activity [46], [48], [49].

We previously reported that the capsid protein associated with the DHBV virions, as well as intracellular mature NCs containing DS DNA, is unphosphorylated, in contrast to immature NCs that consist of heterogeneously phosphorylated core protein [11]. If DHBV, like HBV, secretes empty virions, it would suggest that not all unphosphorylated DHBV capsids are mature and some unphosphorylated DHBV capsids are in fact empty. If all unphosphorylated DHBV capsids do contain DS DNA, it would suggest that DHBV does not secrete empty virions, in contrast to HBV. For the single strand blocking model to accommodate the absence of empty DHBV virions, we suggest that DHBV capsids may package viral pgRNA more efficiently or could package non-specific RNA in host cells, unlike HBV. Consequently, empty DHBV capsids may not be assembled in host cells and thus unavailable for virion formation. Studies are currently underway to determine the relationship between genome content and virion secretion in DHBV.

How SS DNA or RNA triggers the proposed blocking signal is not yet understood. One NC maturation model suggests that the mature NC, by containing DS DNA and thus twice the amount of negative charges as the immature NC containing either the SS DNA or pgRNA, is destabilized as a result of the charge imbalance, triggering an NC conformational change that may be part of the maturation/envelopment signal [50], [51]. In the context of the single strand blocking model, it can be suggested that NC destabilization due to the charge imbalance can not only result from DS DNA accumulation within the maturing NC but also from the lack of any nucleic acid within the empty capsids. Indeed, empty HBV capsids were shown to be unstable especially under low salt conditions [51]. This structural instability may preclude the generation of the blocking signal, thus allowing the secretion of both empty and DS DNA-containing virions. The secretion of SS DNA-containing virions by certain HBV core mutants (the so-called immature secretion mutants) [9], [52], [53] and an avian hepadnavirus (the snowgoose HBV or SGHBV) [54] indicates that the blocking signal may be absent from, or sequestered by, these immature NCs. In addition, low level secretion of SS DNA-containing virions by the WT HBV has also been observed [52], [53] although we saw little, if any, SS DNA in WT HBV virions in our experiments here. It is possible that the stringency with which the single strand blocking signal controls virion morphogenesis may vary, to some extent, with the viral strains, the host cells, and the exact experimental conditions.

In conclusion, we propose that SS DNA or RNA within immature hepadnavirus NCs triggers a blocking signal that negatively regulates their envelopment and secretion, thus ensuring the secretion of only DS DNA (or RNA-DNA hybrid) in virions. However, empty capsids assembled in host cells that are devoid of any viral or cellular nucleic acid, thus lacking the negative signal, can be enveloped and secreted as “empty” virions containing capsids but no nucleic acid. Therefore, the long-sought-for maturation signal, a positive signal for envelopment, may in fact represent the removal of this negative signal, as the maturing NC retains ever-shorter SS DNA due to second (plus-) strand DNA elongation. In common with the maturation signal hypothesis, the SS blocking hypothesis also entails an as yet ill-defined structural change accompanying plus-strand DNA elongation (i.e., loss of the blocking signal). However, the blocking hypothesis predicts that mature, DS DNA-containing NCs share a structural characteristic with empty capsids (both lacking the blocking signal) and furthermore, this characteristic is absent from the pgRNA or SS DNA-containing NCs. The potential pathophysiological significance, if any, of the empty HBV virions remains to be determined. Among other possibilities, these empty virions may function as immune modulators, defective interfering particles, and markers of viral production or tissue damage [13], [55]. As the decision to secrete DNA-filled vs. empty virions is actually made at an earlier step during HBV assembly, i.e., during NC formation and pgRNA packaging (**Figure 8A**), the relative abundance of these two different virion populations reflects the efficiency of pgRNA packaging and may be a convenient marker for monitoring this complex step in the HBV life cycle that requires not only the viral RT and core proteins and pgRNA but also cellular factors [56], [57].

## Methods

### Plasmids

pCMV-HBV contains the HBV (ayw) 1.1-mer over-length genomic sequence driven by the cytomegalovirus (CMV) immediate-early promoter [38]. pCMV-HBV-Pol^-^ and pCMV-HBV-Env^-^ were derived from pCMV-HBV and are defective in polymerase and envelope protein expression, respectively [26]. pCMV-HBV-Core^-^Pol^-^ was derived from pCMV-HBV-Pol^-^ by introducing a 4 nt (GATC) insertion at position 1986 creating a frameshift mutation after codon 30 in the core gene. pCMV-HBV-Y63D bears a Y63D substitution in the RT gene eliminating DNA synthesis [22]. All site-specific mutations were confirmed by automated DNA sequencing. pA3G-Flag expresses the Flag-tagged human Apobec3G protein [18].

### Transient transfection and stably transfected cell line

The human hepatoma cell line HepG-2 were transfected by FuGene 6 (Roche) [18], [58]. The HepAD38 cell line was derived from HepG-2 cell and replicates HBV in a tetracycline (Tet)-repressible manner [59].

### Analyses of viral core protein expression and DNA synthesis

Core protein expression levels in the cytoplasmic lysate were analyzed by SDS-PAGE and western blotting using the anti-HBV core antibody (Dako, a rabbit polyclonal antibody; or a mouse monoclonal specific for the N-terminal end of the core protein [23]) as described previously [18], [58]. Briefly, primary antibodies were diluted at 1∶1,000 and incubated with proteins bound to Immobilon-P membrane (Millipore) overnight. Secondary anti-mouse or anti-rabbit peroxidase labeled antibodies were used at 1∶20,000 dilution. Chemilluminescence was used for detection of the bound antibody. A rabbit polyclonal antibody specific for the last 14 residues of the HBV core protein [39] was used where indicated to specifically detect the C-terminal sequence of the core protein. Virion- or naked capsid-associated DNA was isolated and analyzed by Southern blotting as described previously [18], [58].

### Native agarose gel analysis of capsids and capsid-associated nucleic acid

Native HBV NCs were purified from transfected HepG-2 cells by sucrose gradient centrifugation [11], [60]. Recombinant HBV capsids purified from bacteria were obtained from Virogen. Purified NCs and capsids were resolved by native agarose gel electrophoresis [18], [58], [61], [62] and the gel was stained by SYBR Gold (Invitrogen) to detect RNA and DNA; after destaining with 10% methanol and 7% glacial acetic acid for 2 hr, the same gel was restained with SYPRO Ruby (Sigma) to detect proteins. The protein and nucleic acid signals were detected and quantified by using a Molecular Imager (BioRad FX-PRO Plus).

### Analysis of virion secretion and CsCl density gradient centrifugation

Culture medium containing HBV virions and naked NCs was concentrated by polyethylene glycol precipitation and digested with DNase I (1 mg/ml at 37°C for 1 h) to eliminate residual plasmid DNA and fractionated by isopycnic CsCl gradient ultracentrifugation [7], [11], [61] to remove naked (non-enveloped) NCs. Purified virion fractions or DNase digested concentrated medium samples were analyzed by native agarose gel electrophoresis as described [63]. Encapsidated RNA or DNA in viral particles was detected using ^32^P-labeled RNA or DNA probe as indicated, followed by detection of core proteins associated with virions or naked NCs on the same membrane using the anti-core antibody. Goat polyclonal anti-HBV surface protein (Dako) was then used to detect the viral envelope proteins after stripping the membrane. The nature of virion DNA was determined by Southern blotting after extraction from purified virions using SDS/proteinase K digestion [7], [11]. The core protein associated with virions was also analyzed by SDS-PAGE and western blot analysis as described above. Sera from HBV infected chimpanzees have been described before [24]. DNA levels were quantified using phosphoimaging following Southern blot analysis and protein levels using densitometry following western blot analysis. Viral DNA and core protein standards were used to generate standard curves from which sample virion DNA and core protein levels were quantified. To estimate the levels of HBsAg, purified recombinant HBsAg (eEnzyme) was used as a quantitative standard. Following SDS-PAGE and western transfer, the HBV envelope proteins were detected by a polyclonal rabbit anti-HBs antibody (Virostat). To prepare virion-derived capsids for EM, the virion fractions were treated with 1% NP40-10 mM dithiothreitol (DTT) for 30 minutes on ice to remove the viral envelope and release the capsids [12], [14]. The released virion capsids, along with complete virions were observed under EM after neagative staining as described below.

### EM and 3-D image reconstruction

For each sample, an aliquot of 3 µl was placed on a freshly glow-discharged continuous carbon coated copper grid. Phosphotungstic acid negative stain was applied by standard drop method and the sample was examined in JEOL 1400 TEM at 120 kV. For the 3-D reconstruction, 10–20 micrographs were collected with a Gatan Orius SC1000 CCD camera with Digital Micrograph at a calibrated magnification of 22,510X and 27,845X. Viral particles were selected and processed using Robem [64]. The reconstruction processes were performed without CTF correction using icosahedral averaging with the program Auto3dem, which generated a random model directly from the raw data as the initial starting structure [64]. The final resolution was determined where the Fourier shell correlation fell below 0.5 as reported in the summary output file of Auto3dem. The number of viral particles selected, the final resolution and diameters of the 3-D reconstructions are listed in **Table S1**. The final pixel sizes were 1.26 for the WT HBV capsids and 2.52 for all other constructs. The final reconstructions were colored radially using the program Chimera [65].

## Supporting Information

Figure S1 **Quantification of CsCl density profiles of HBV virions and naked capsids.** The relative levels of HBV DNA, RNA, core protein, and surface proteins associated with the virions or naked capsids in the culture medium of HepG-2 cells transfected with the WT (top), Y63D (middle), or Pol- (bottom) HBV constructs were plotted across the CsCl density. Note that the surface proteins were only detected in virion fractions and the viral RNA only in naked capsid fractions. The numerals denote the peak densities of DNA-filled virions (1), empty virions (2), DNA or RNA filled naked capsids (3), or empty capsids (4). The Y-axis represents percent of the total virion or naked capsid signal in each virion or capsid fraction. Virion and naked capsid fractions were quantified separately due to the lower overall virion signals as compared to naked capsids.
(TIF)

Figure S2 **Southern blot analysis of HBV DNA associated with virions and naked capsids fractionated by CsCl gradient centrifugation.** pCMV-HBV was transfected into HepG-2 cells and viral particles concentrated from the culture medium were fractionated by CsCl gradient centrifugation. Individual fractions were treated with SDS/proteinase K to release the viral DNA, which was then resolved by agarose gel electrophoresis and detected by Southern blotting using an HBV DNA probe. RC, relaxed circular DNA; DSL, double-stranded linear DNA; SS, single-stranded DNA.
(TIF)

Figure S3 **Lack of virion secretion by the HBV mutant defective in envelope protein expression by CsCl density gradient analysis.** The envelope-deficient mutant HBV genome was transfected into HepG-2 cells and viral particles concentrated from the culture media as in described **Figure 1**. The concentrated media were then fractionated by CsCl gradient centrifugation. Gradient fractions were analyzed by resolving viral particles on native agarose gels. HBV DNA (top) and core protein (bottom) were detected as described in **Figure 1**. The numbered fractions mark the DNA-containing (#1) or DNA-free (#2) virion peaks (both absent from this mutant), and the naked capsid peaks containing viral DNA or RNA (#3) or no nucleic acid (empty, #4), with their respective densities indicated in bold at the bottom. The diagrams on the side depict the structures of the capsids as described in **Figure 1**.
(TIF)

Figure S4 **Similar reactivity of HBV capsids and core subunits with two different anti-HBc antibodies.** The virion fractions isolated from the culture medium of WT HBV-transfected HepG-2 cells by CsCl gradient fractionation were resolved by native agarose gel electrophoresis (**A**) or SDS-PAGE (**B**). The HBV core protein was detected using the mouse monoclonal (top) or the rabbit polyclonal antibody (**A**, middle; **B**, bottom). The viral DNA was detected by reprobing the same membrane that was used for core protein detection by using an HBV DNA probe (**A**, bottom). The direction of centrifugation is indicated by the arrow. The virion core protein peak (containing mostly empty virions) is shown in lane 2 and the virion DNA peak is in lane 3. The fraction shown in lane 1 contained little DNA and contained almost entirely empty virions.
(TIF)

Figure S5 **Analysis of HBV virions in chimpanzee sera before and after CsCl gradient fractionation.** **A**. Two HBV positive, (+), chimpanzee serum samples (chimpanzee 1616 at week 20 and 23 post-infection or PI; lanes 1–2) and four HBV negative, (-), serum samples (from the same four chimpanzees shown in **Figure 4** but before HBV infection; lanes 3–6) were resolved by agarose gel electrophoresis (top) or SDS-PAGE (bottom) and the viral core protein was detected by western blotting using the anti-HBV core antibody. **B**. HBV virions in the week 7 PI serum from chimpanzee A0A006 were fractionated by CsCl gradient ultracentrifugation and the individual fractions (lanes 1–6), along with the crude serum input (lane 8) and the pre-infection serum (lane 7) from the same chimpanzee were analyzed by agarose gel electrophoresis and Southern blotting to detect viral DNA (top) or by SDS-PAGE followed by western blotting to detect the viral core protein (bottom). Intracellular (IC) HBV capsids purified from HBV transfected HepG-2 cells were loaded in lane 9 (bottom) as a control. V, virion; C, core protein. Note the lack of cross-reactivity of the anti-core antibody to the pre-infection serum samples by either the agarose gel or SDS-PAGE analysis (**A** & **B**). The direction of centrifugation is indicated by the arrow.
(TIF)

Figure S6 **Estimatation of HBsAg levels in the sera of HBV infected chimpanzees.** **A**. The levels of HBsAg from the indicated amounts of the week 14 post-infection (lanes 1–3) or pre-infection (lanes 4–6) serum from chimpanzee 1618 were estimated by comparison with a dilution series of HBsAg standard (lane 7–11, containing the small surface protein or p24, the most abundant of the three viral envelope proteins; eEnzyme). The samples were resolved by SDS-PAGE and HBsAg was detected by western blotting using the rabbit anti-HBs antibody (Virostat) able to recognize denatured surface proteins on SDS-PAGE as well as HBsAg particles resolved on agarose gels. SDS-PAGE was used due to the uncertain nature of the oligomeric state of the HBsAg standard. The amount of HBsAg (mainly the two small surface proteins p24 and gp27) in the HBV positive serum was estimated to be 1.17 mg/ml. p24 and gp27, unmodified and glycosylated small surface protein; gp33 and gp36, singly- and doubly-glycosylated middle surface protein [27]. The large surface proteins (p39 and gp42) were not definitively identified due to their low abundance. The protein molecular weight markers (M) are indicated on the right. **B**. The amounts of HBsAg in the other chimpanzee serum samples were estimated by comparison with the serum sample shown in lane 4 whose HBsAg concentration was predetermined in panel **A** (1.17 mg/ml or 1170 ng/µl). The serum sample (1 µl per lane, loaded in the same order as in **Figure 4**, lanes 1–7) were resolved by native agarose gel electrophoresis and detected by western blotting. The titers of HBsAg in the serum samples were calculated from the estimated HBsAg concentrations assuming 100 copies of surface protein per HBsAg particle. The concentrations of the virions in the same serum samples were calculated using the estimated virion core protein amounts shown in **Figure 4** assuming 240 copies of core protein per capsid (virion).
(TIF)

Figure S7 **Detection of DNA and RNA on agarose gel by SYBR Gold staining.** The indicated amounts of yeast tRNA and a 1 kb DNA m. w. marker (NEB) were resolved on an agarose gel and detected by SYBR Gold staining. Note that the 3 kb DNA represents 25%, and the other DNA species, 8%, of the total DNA loaded. The detection limit was approximately 300 pg for tRNA and 40 pg for the 3 kb DNA.
(TIF)

Figure S8 **Nucleic acid and protein staining of purified HBV capsids.** WT HBV NCs (lanes 10, 11) and HBV capsids devoid of the polymerase (Pol^-^) (lane 1) were purified from HepG-2 cells transfected with pCMV-HBV and pCMV-HBV-Pol^-^, respectively. WT HBV capsids purified from E. Coli (lanes 2–9) were purchased from Virogen. The capsids were resolved by native agarose gel electrophoresis. Capsid-associated nucleic acid was detected by SYBR Gold staining (top) and the capsid proteins were detected by destaining of the SYBR Gold signal and subsequent restaining of the same gel with SYPRO Ruby (bottom). C, capsids.
(TIF)

Figure S9 **3-D image reconstruction of HBV capsids.** Viral capsid structures were reconstructed from negatively stained particles of E. Coli-derived full-length and the C-terminally truncated (ΔCTD) capsids, as well as WT and Pol^-^ HBV capsids purified from transfected HepG-2 cells. The top row shows the surface renderings of the reconstructions and the bottom shows cross-sections. All models are depicted at 1 σ. Color key depicts color range according to radial distance in angstrom.
(TIF)

Figure S10 **WT and Pol- HBV genomes expressed HBV core protein with complete C-terminal sequence in transfected cells.** WT HBV NCs (lanes 3 & 7) and HBV capsids devoid of the polymerase (Pol-) (lanes 4 & 8) were isolated from HepG-2 cells transfected with pCMV-HBV and pCMV-HBV-Pol-, respectively. Full-length (FL; lanes 1 & 5) and C-terminally truncated (at residue 144, ΔCTD; lanes 2 & 6) HBV capsids purified from E. Coli were from Virogen. The core proteins were resolved by SDS-PAGE and after transfer to membrane, detected by sequential probing first with a rabbit polyclonal antibody against the last 14 residues of the core protein (anti-CTD, lanes 1–4) and then with a mouse monoclonal antibody against the N-terminal core sequence (anti-NTD, lanes 5–8). C, full-length core protein; ΔCTD, C-terminally truncated core protein.
(TIF)

Table S1 **Overview of the 3-D reconstruction results.**
(DOC)
