# Supplementary figures and images for: Secretion of Genome-Free Hepatitis B Virus – Single Strand Blocking Model for Virion Morphogenesis of Para-retrovirus
Source: PLoS Pathog. 2011 Sep 22;7(9):e1002255. doi: 10.1371/journal.ppat.1002255 (PMC3178560; doi:10.1371/journal.ppat.1002255)

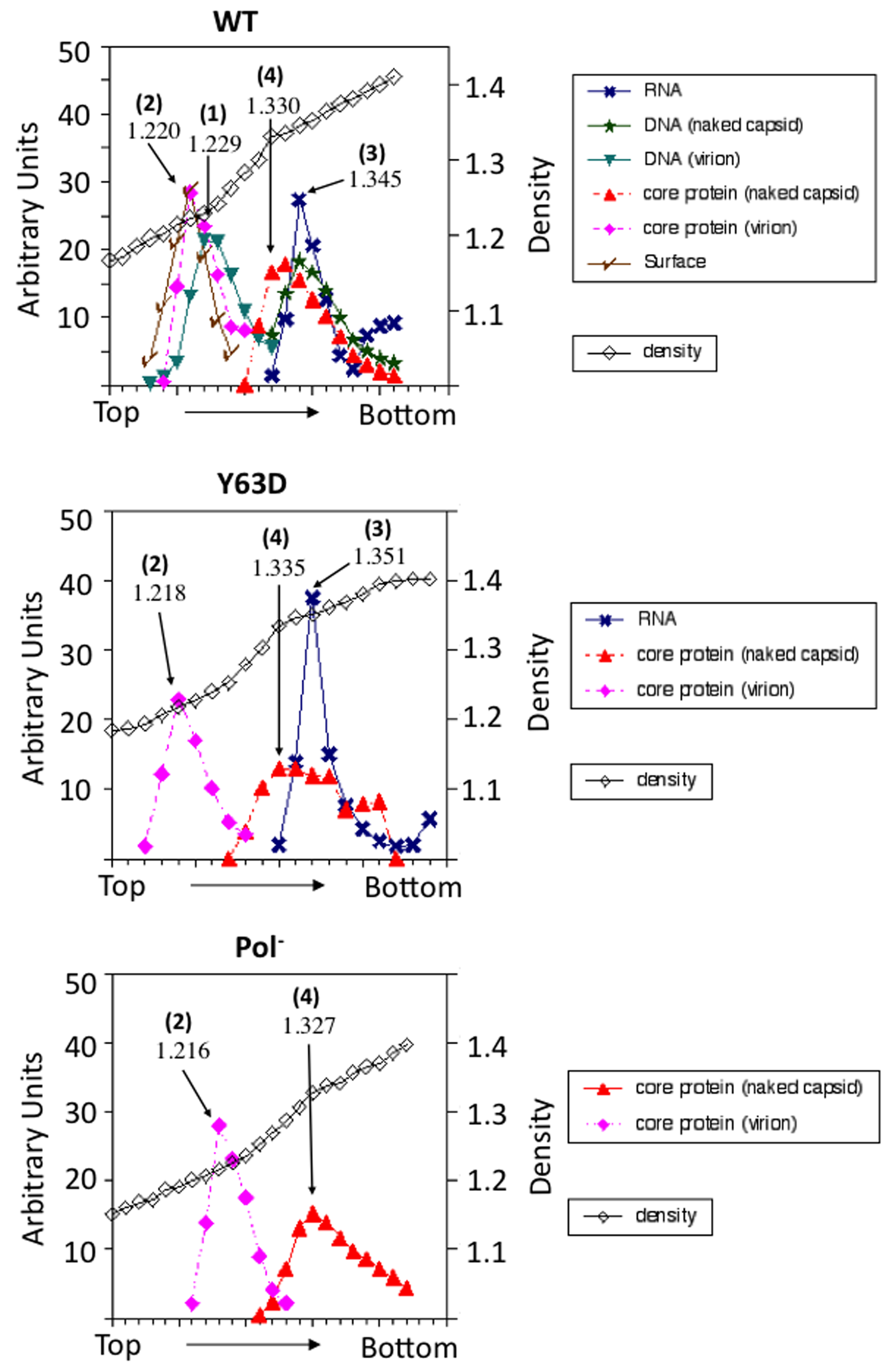

Supplement: Figure S1 — Quantification of CsCl density profiles of HBV virions and naked capsids. The relative levels of HBV DNA, RNA, core protein, and surface proteins associated with the virions or naked capsids in the culture medium of HepG-2 cells transfected with the WT (top), Y63D (middle), or Pol- (bottom) HBV constructs were plotted across the CsCl density. Note that the surface proteins were only detected in virion fractions and the viral RNA only in naked capsid fractions. The numerals denote the peak densities of DNA-filled virions (1), empty virions (2), DNA or RNA filled naked capsids (3), or empty capsids (4). The Y-axis represents percent of the total virion or naked capsid signal in each virion or capsid fraction. Virion and naked capsid fractions were quantified separately due to the lower overall virion signals as compared to naked capsids. (TIF) [file ppat.1002255.s001.tif]

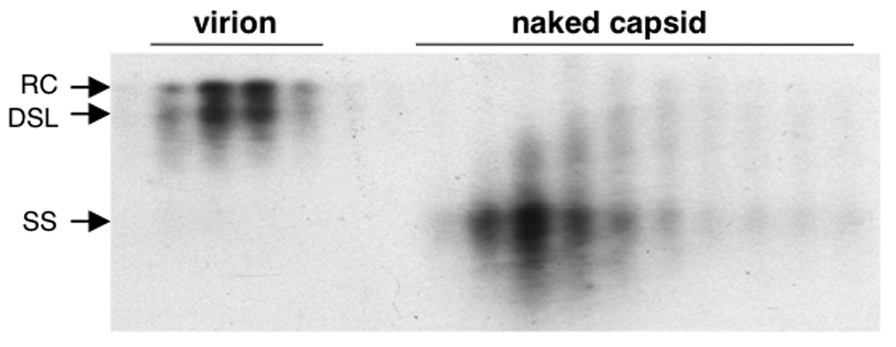

Supplement: Figure S2 — Southern blot analysis of HBV DNA associated with virions and naked capsids fractionated by CsCl gradient centrifugation. pCMV-HBV was transfected into HepG-2 cells and viral particles concentrated from the culture medium were fractionated by CsCl gradient centrifugation. Individual fractions were treated with SDS/proteinase K to release the viral DNA, which was then resolved by agarose gel electrophoresis and detected by Southern blotting using an HBV DNA probe. RC, relaxed circular DNA; DSL, double-stranded linear DNA; SS, single-stranded DNA. (TIF) [file ppat.1002255.s002.tif]

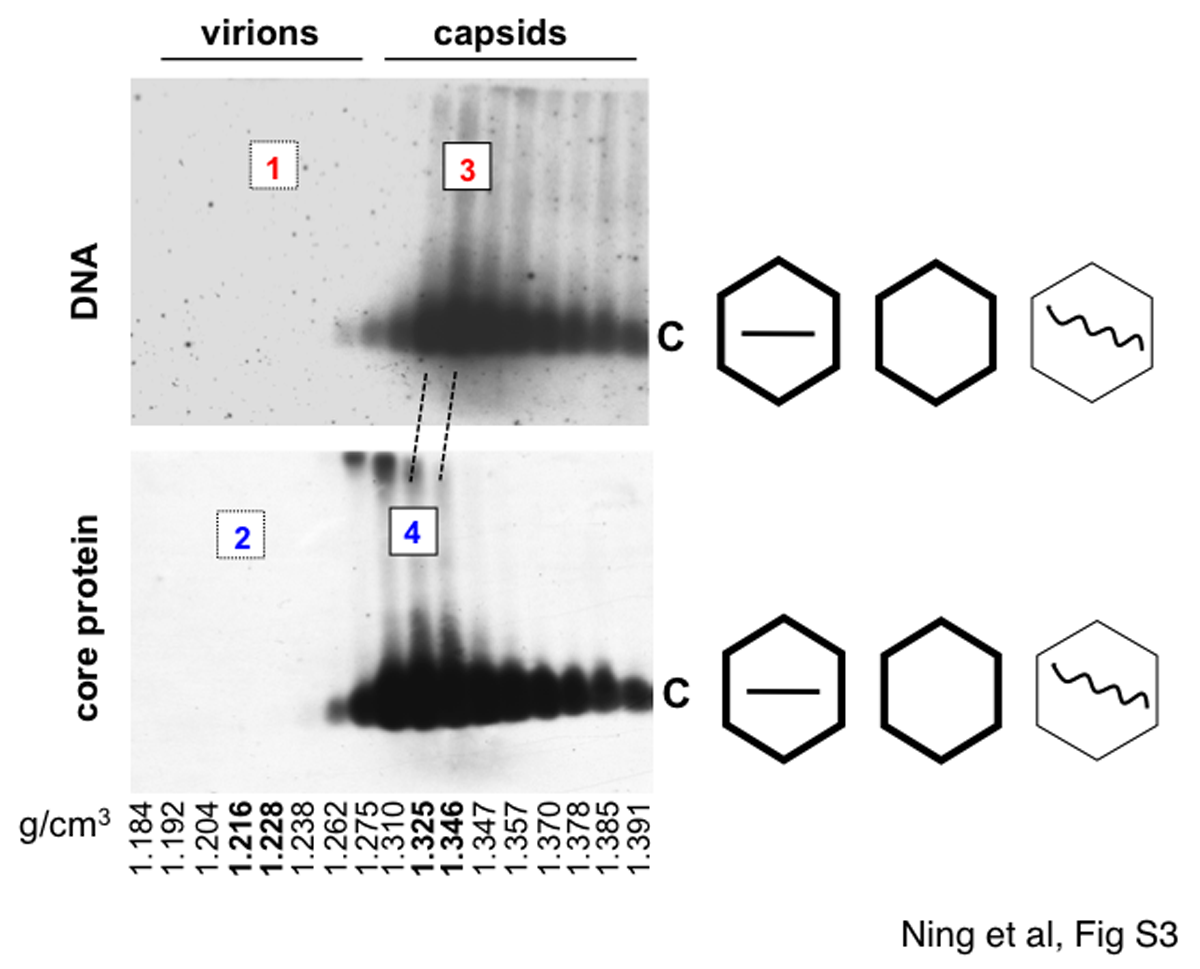

Supplement: Figure S3 — Lack of virion secretion by the HBV mutant defective in envelope protein expression by CsCl density gradient analysis. The envelope-deficient mutant HBV genome was transfected into HepG-2 cells and viral particles concentrated from the culture media as in described Figure 1. The concentrated media were then fractionated by CsCl gradient centrifugation. Gradient fractions were analyzed by resolving viral particles on native agarose gels. HBV DNA (top) and core protein (bottom) were detected as described in Figure 1. The numbered fractions mark the DNA-containing (#1) or DNA-free (#2) virion peaks (both absent from this mutant), and the naked capsid peaks containing viral DNA or RNA (#3) or no nucleic acid (empty, #4), with their respective densities indicated in bold at the bottom. The diagrams on the side depict the structures of the capsids as described in Figure 1. (TIF) [file ppat.1002255.s003.tif]

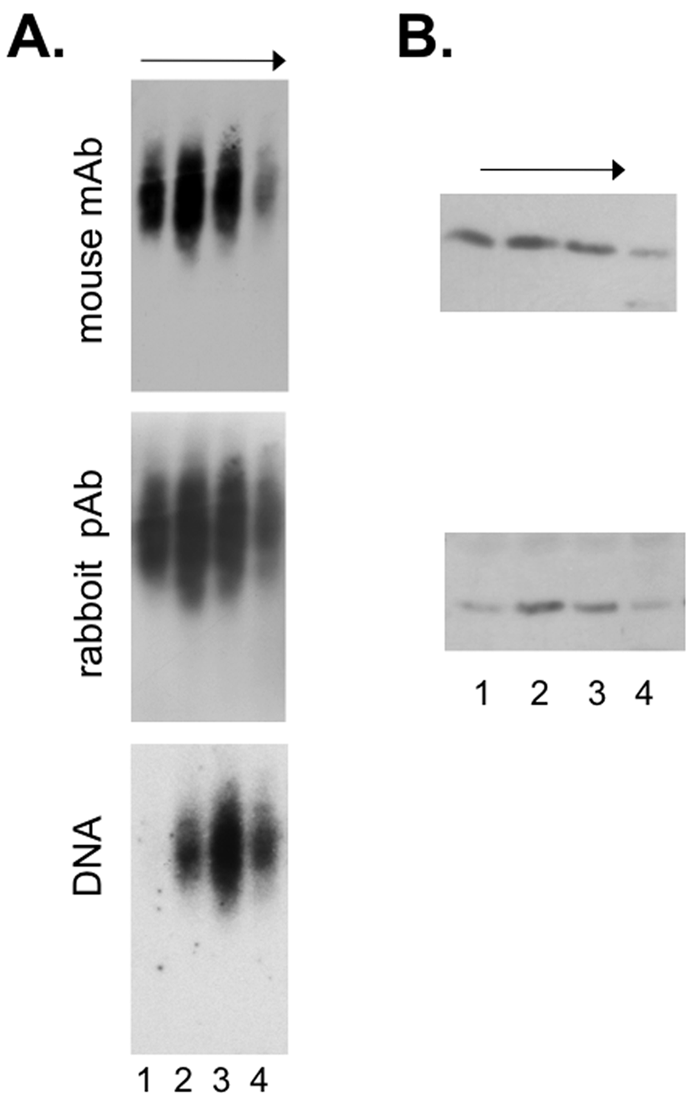

Supplement: Figure S4 — Similar reactivity of HBV capsids and core subunits with two different anti-HBc antibodies. The virion fractions isolated from the culture medium of WT HBV-transfected HepG-2 cells by CsCl gradient fractionation were resolved by native agarose gel electrophoresis (A) or SDS-PAGE (B). The HBV core protein was detected using the mouse monoclonal (top) or the rabbit polyclonal antibody (A, middle; B, bottom). The viral DNA was detected by reprobing the same membrane that was used for core protein detection by using an HBV DNA probe (A, bottom). The direction of centrifugation is indicated by the arrow. The virion core protein peak (containing mostly empty virions) is shown in lane 2 and the virion DNA peak is in lane 3. The fraction shown in lane 1 contained little DNA and contained almost entirely empty virions. (TIF) [file ppat.1002255.s004.tif]

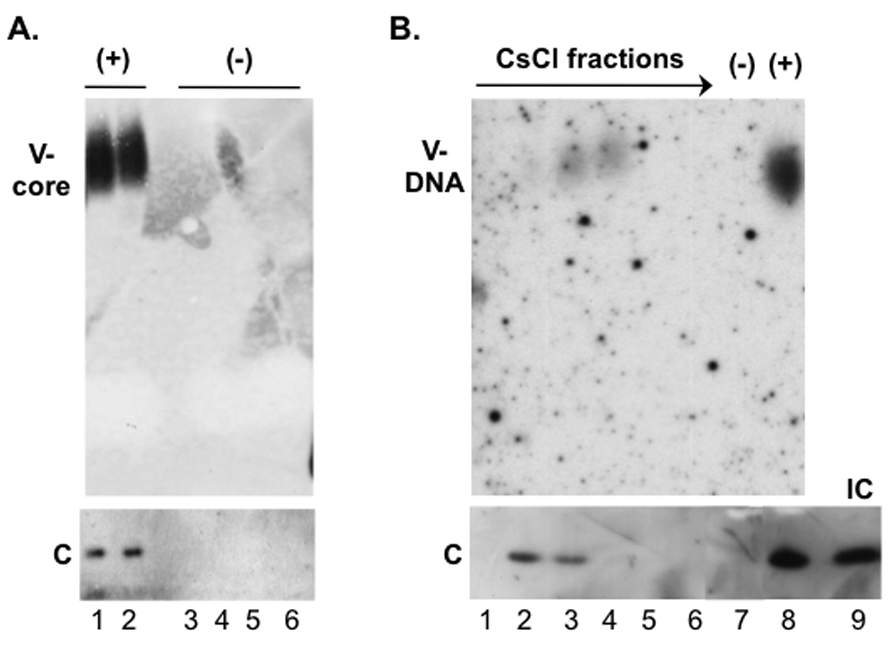

Supplement: Figure S5 — Analysis of HBV virions in chimpanzee sera before and after CsCl gradient fractionation. A. Two HBV positive, (+), chimpanzee serum samples (chimpanzee 1616 at week 20 and 23 post-infection or PI; lanes 1–2) and four HBV negative, (-), serum samples (from the same four chimpanzees shown in Figure 4 but before HBV infection; lanes 3–6) were resolved by agarose gel electrophoresis (top) or SDS-PAGE (bottom) and the viral core protein was detected by western blotting using the anti-HBV core antibody. B. HBV virions in the week 7 PI serum from chimpanzee A0A006 were fractionated by CsCl gradient ultracentrifugation and the individual fractions (lanes 1–6), along with the crude serum input (lane 8) and the pre-infection serum (lane 7) from the same chimpanzee were analyzed by agarose gel electrophoresis and Southern blotting to detect viral DNA (top) or by SDS-PAGE followed by western blotting to detect the viral core protein (bottom). Intracellular (IC) HBV capsids purified from HBV transfected HepG-2 cells were loaded in lane 9 (bottom) as a control. V, virion; C, core protein. Note the lack of cross-reactivity of the anti-core antibody to the pre-infection serum samples by either the agarose gel or SDS-PAGE analysis (A & B). The direction of centrifugation is indicated by the arrow. (TIF) [file ppat.1002255.s005.tif]

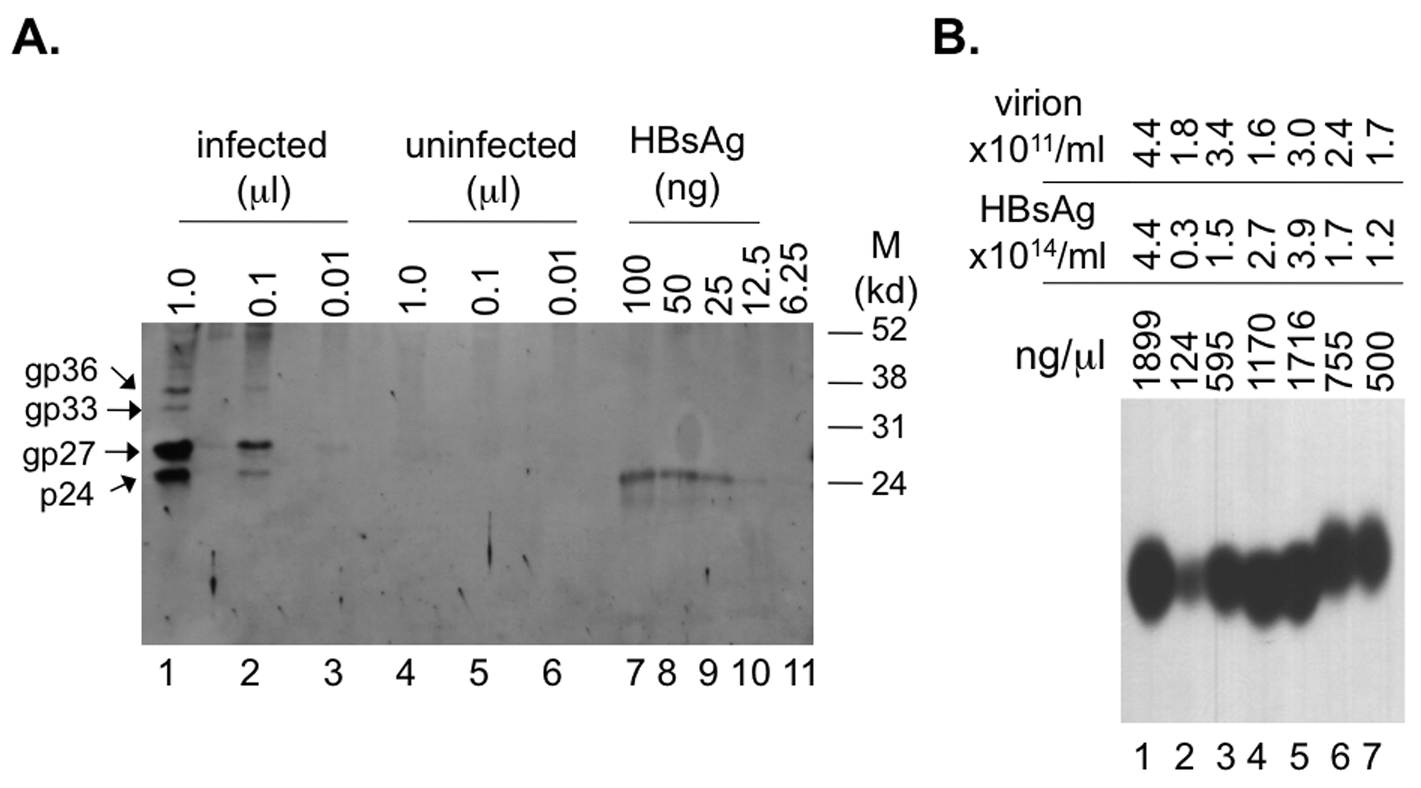

Supplement: Figure S6 — Estimatation of HBsAg levels in the sera of HBV infected chimpanzees. A. The levels of HBsAg from the indicated amounts of the week 14 post-infection (lanes 1–3) or pre-infection (lanes 4–6) serum from chimpanzee 1618 were estimated by comparison with a dilution series of HBsAg standard (lane 7–11, containing the small surface protein or p24, the most abundant of the three viral envelope proteins; eEnzyme). The samples were resolved by SDS-PAGE and HBsAg was detected by western blotting using the rabbit anti-HBs antibody (Virostat) able to recognize denatured surface proteins on SDS-PAGE as well as HBsAg particles resolved on agarose gels. SDS-PAGE was used due to the uncertain nature of the oligomeric state of the HBsAg standard. The amount of HBsAg (mainly the two small surface proteins p24 and gp27) in the HBV positive serum was estimated to be 1.17 mg/ml. p24 and gp27, unmodified and glycosylated small surface protein; gp33 and gp36, singly- and doubly-glycosylated middle surface protein [27]. The large surface proteins (p39 and gp42) were not definitively identified due to their low abundance. The protein molecular weight markers (M) are indicated on the right. B. The amounts of HBsAg in the other chimpanzee serum samples were estimated by comparison with the serum sample shown in lane 4 whose HBsAg concentration was predetermined in panel A (1.17 mg/ml or 1170 ng/µl). The serum sample (1 µl per lane, loaded in the same order as in Figure 4, lanes 1–7) were resolved by native agarose gel electrophoresis and detected by western blotting. The titers of HBsAg in the serum samples were calculated from the estimated HBsAg concentrations assuming 100 copies of surface protein per HBsAg particle. The concentrations of the virions in the same serum samples were calculated using the estimated virion core protein amounts shown in Figure 4 assuming 240 copies of core protein per capsid (virion). (TIF) [file ppat.1002255.s006.tif]

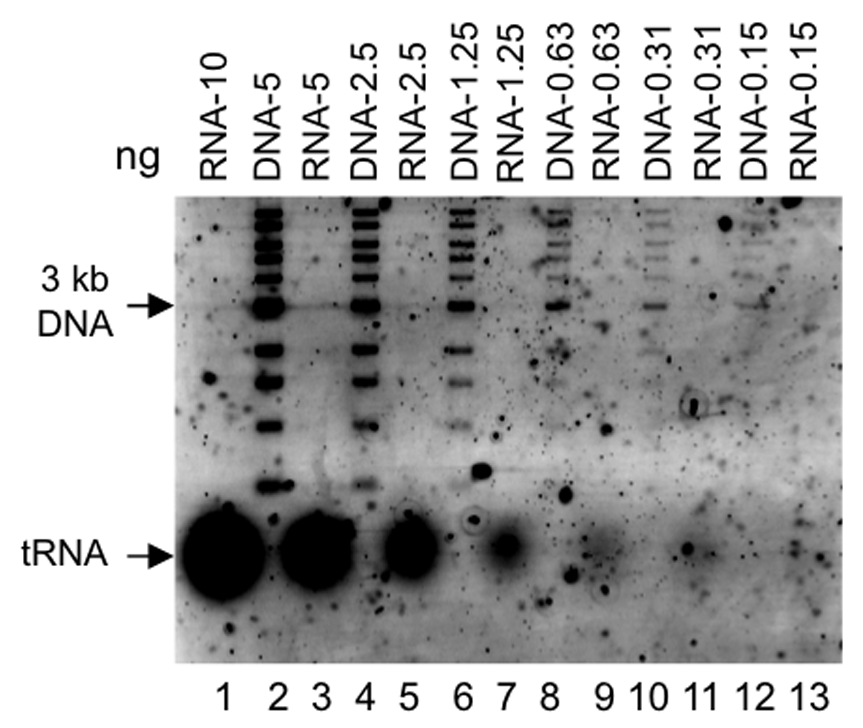

Supplement: Figure S7 — Detection of DNA and RNA on agarose gel by SYBR Gold staining. The indicated amounts of yeast tRNA and a 1 kb DNA m. w. marker (NEB) were resolved on an agarose gel and detected by SYBR Gold staining. Note that the 3 kb DNA represents 25%, and the other DNA species, 8%, of the total DNA loaded. The detection limit was approximately 300 pg for tRNA and 40 pg for the 3 kb DNA. (TIF) [file ppat.1002255.s007.tif]

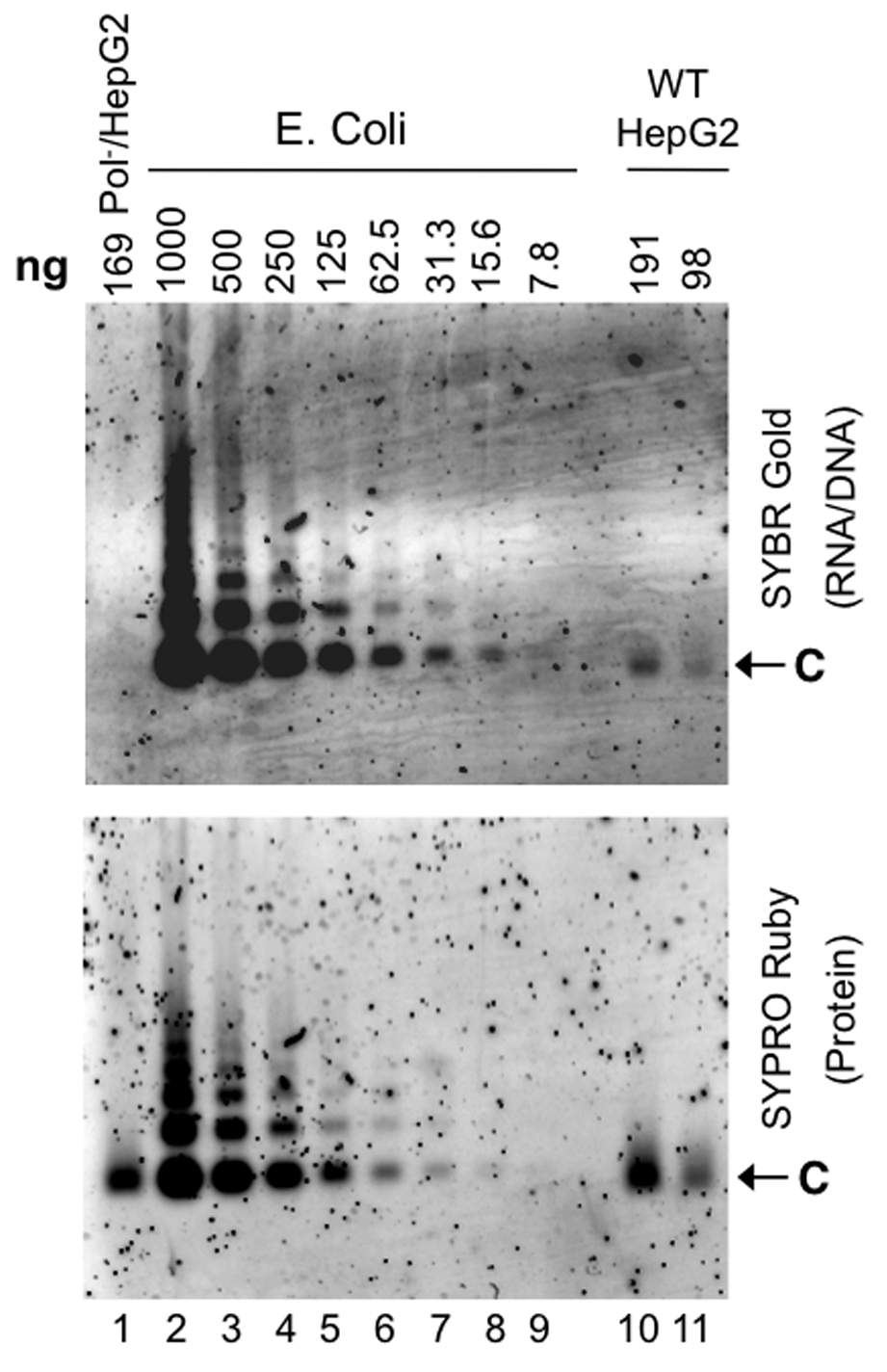

Supplement: Figure S8 — Nucleic acid and protein staining of purified HBV capsids. WT HBV NCs (lanes 10, 11) and HBV capsids devoid of the polymerase (Pol-) (lane 1) were purified from HepG-2 cells transfected with pCMV-HBV and pCMV-HBV-Pol-, respectively. WT HBV capsids purified from E. Coli (lanes 2–9) were purchased from Virogen. The capsids were resolved by native agarose gel electrophoresis. Capsid-associated nucleic acid was detected by SYBR Gold staining (top) and the capsid proteins were detected by destaining of the SYBR Gold signal and subsequent restaining of the same gel with SYPRO Ruby (bottom). C, capsids. (TIF) [file ppat.1002255.s008.tif]

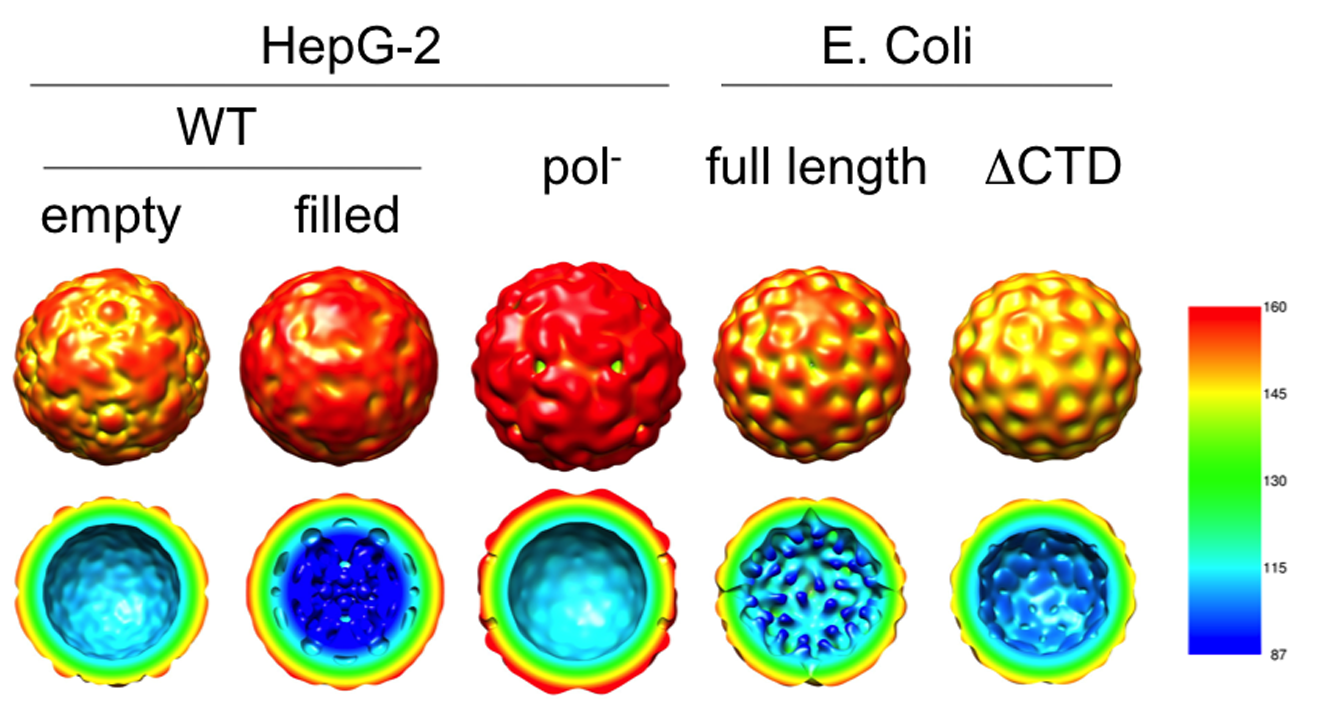

Supplement: Figure S9 — 3-D image reconstruction of HBV capsids. Viral capsid structures were reconstructed from negatively stained particles of E. Coli-derived full-length and the C-terminally truncated (ΔCTD) capsids, as well as WT and Pol- HBV capsids purified from transfected HepG-2 cells. The top row shows the surface renderings of the reconstructions and the bottom shows cross-sections. All models are depicted at 1 σ. Color key depicts color range according to radial distance in angstrom. (TIF) [file ppat.1002255.s009.tif]

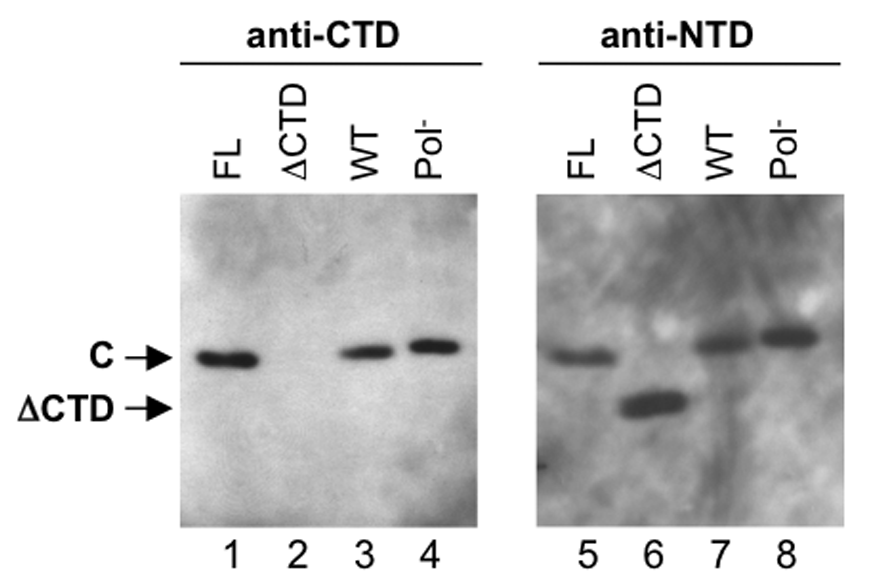

Supplement: Figure S10 — WT and Pol- HBV genomes expressed HBV core protein with complete C-terminal sequence in transfected cells. WT HBV NCs (lanes 3 & 7) and HBV capsids devoid of the polymerase (Pol-) (lanes 4 & 8) were isolated from HepG-2 cells transfected with pCMV-HBV and pCMV-HBV-Pol-, respectively. Full-length (FL; lanes 1 & 5) and C-terminally truncated (at residue 144, ΔCTD; lanes 2 & 6) HBV capsids purified from E. Coli were from Virogen. The core proteins were resolved by SDS-PAGE and after transfer to membrane, detected by sequential probing first with a rabbit polyclonal antibody against the last 14 residues of the core protein (anti-CTD, lanes 1–4) and then with a mouse monoclonal antibody against the N-terminal core sequence (anti-NTD, lanes 5–8). C, full-length core protein; ΔCTD, C-terminally truncated core protein. (TIF) [file ppat.1002255.s010.tif]
