# Supplementary material for: Secretion of Genome-Free Hepatitis B Virus – Single Strand Blocking Model for Virion Morphogenesis of Para-retrovirus
Source: PLoS Pathog. 2011 Sep 22;7(9):e1002255. doi: 10.1371/journal.ppat.1002255 (PMC3178560; doi:10.1371/journal.ppat.1002255)
Supplement: Table S1 — Overview of the 3-D reconstruction results. (DOC) [file ppat.1002255.s011.doc]

**Table S1. Overview of the 3-D reconstruction results**

| **Types of capsids**^a^ | **Number of selected capsids** | **Resolution (Å)** | **Diameter (nm)** |
| --- | --- | --- | --- |
| WT (full) | 20 | 46.5 | 33.6 |
| WT (empty) | 133 | 32.8 | 33.6 |
| Pol^-^ | 124 | 40.0 | 34.5 |
| Full-length | 238 | 34.3 | 32.6 |
| Truncated | 470 | 33.5 | 32.6 |

^a^WT and pol^-^ capsids were purified from transfected HepG-2 cells. Full-length and C-terminally truncated (at position 144) capsids were recombinant capsids purified from E. Coli.
